# Nature immersion in virtual reality has limited hypoalgesia effects for muscle pain or motor performance benefits during prescribed and self-regulated tasks

**DOI:** 10.1007/s00421-026-06399-z

**Published:** 2026-08-19

**Authors:** Callum A. O’Malley, David J. Harris, Sam W. Hughes, Jack E. Cottrell, Daniel Lloyd, Samuel J. Vine, Tom Arthur

**Affiliations:** 1 Department of Public Health and Sport Sciences, University of Exeter, St Luke’s Campus, Exeter, EX1 2LU UK; 2 Exeter Medical School, University of Exeter, Exeter, EX1 2LU UK

**Keywords:** Naturally occurring muscle pain, Virtual reality, Effort, Motor behaviour, Psychophysiology

## Abstract

**Purpose:**

Virtual reality (VR) reduces experimental and clinical pain, but whether these effects extend to naturally occurring muscle pain (NOMP) during exercise remains unclear. This study examined whether immersive nature-based VR attenuates NOMP and improves motor performance during prescribed and self-regulated exercise.

**Methods:**

Two within-participant crossover studies were conducted involving 12 (Study 1) and 24 (Study 2) healthy participants. In Study 1, participants completed a stepped incremental cycling task to exhaustion. In Study 2, participants performed self-regulated intermittent isometric handgrip contractions at light (13/100) and strong (50/100) perceived effort. Tasks were completed under immersive VR (nature scene), two-dimensional sham, and no-intervention control conditions in pseudo-randomised, counterbalanced orders. Pain onset, NOMP intensity, perceived effort, and time to exhaustion were assessed in Study (1) Muscle pain intensity and force production were measured in Study (2) Repeated measures ANOVAs and assessed main condition effects.

**Results:**

Immersive VR did not delay pain onset * (p=.159)*, prolong time to exhaustion * (p=.204)*, or lower perceived effort compared within sham or control conditions $\:({p}^{{\prime\:}}s>.215)$. A small, yet practically meaningful, reduction in NOMP intensity occurred early during cycling $\:({F}_{\mathrm{2,22}}=4.518,p=.023,\:{\eta\:}_{p}^{2}=0.291[\mathrm{0.002,0.500}\left]\right)$ but was not sustained at later phases of the task $\:({p}^{{\prime\:}}s<.369)$. In Study 2, NOMP intensity * (p=.815)* and force production measures $\:({p}^{{\prime\:}}s<.325)$ were similar across conditions.

**Conclusion:**

Nature-based immersive VR had limited effects on NOMP and motor performance. Pain types that are peripherally driven, like NOMP, may be less affected by VR interventions which target central mechanisms of pain modulation. Thus, hypoalgesia effects with VR are potentially pain-type dependent.

**Supplementary Information:**

The online version contains supplementary material available at https://doi.org/10.1007/s00421-026-06399-z.

## Introduction

Naturally occurring muscle pain (NOMP) is an inherent feature of physical tasks (O’Connor and Cook 1999). Emanating from the stimulation of metabo- and mechano-sensitive nociceptors in the working musculature (Graven-Nielsen 2006; Mense 2003), nociceptive signals are relayed through afferent fibres to the brain where they are processed into NOMP (Hofbauer et al. 2001). Increased exercise intensity often results in greater nociception and intensified NOMP (Cook et al. 1997). Accordingly, NOMP is considered acute, transient, and a signal of potential tissue damage (O’Malley et al. 2024).

During fixed workload tasks (e.g., constant force), individuals can maintain task performance in the presence of NOMP by increasing the engagement of physical and cognitive resources (i.e., its effort) (Gendolla et al. 2019; Halperin and Vigotsky 2024). Therefore, task performance can be maintained but is associated with a higher perceived effort (Norbury et al. 2022). However, little is known about the effects of NOMP during self-regulated tasks such as exercising at a constant perceived effort intensity. Theoretically, physical outputs (e.g., force) should decrease with increasing NOMP intensity due to neurophysiological and cognitive effects which make the task more effortful for a given output (Rohel et al. 2021; Sanderson et al. 2021). As such, the onset and tolerance of NOMP is a strong influence on motor task performance regardless of task type (Cook et al. 1997; O’Connor and Cook 1999). Consequently, exploring ways that an individual can control and manipulate pain experiences to enhance task performance is an intriguing prospect, especially as NOMP is ever-present in daily life (O’Malley et al. 2024).

Previous research has used a variety of neuromodulation techniques including electrical (Angius et al. 2016) or magnetic (Tamura et al. 2004) brain stimulations to reduce pain intensity (hypoalgesia). However, immersive virtual reality could be a novel and less aversive method to reduce pain (Medina et al. 2024). Trials exposing participants to nature scenes in virtual reality show higher pain thresholds (i.e., pain onset), increased pain tolerance (i.e., a maximal point participant[s] can deal with pain), and an overall hypoalgesia effect compared to no intervention (Hoffman et al. 2004a, b; Hughes et al. 2019). Moreover, the more present individuals feel within virtual reality, the greater the hypoalgesia effect (Hoffman et al. 2004a, b). It is likely the distracting visual and audio cues presented in virtual reality reduce the activity of pain-related brain areas like the anterior cingulate cortex, insula, and thalamus from processing pain-generating signals (Medina et al. 2024; Mehesz et al. 2021; Steininger et al. 2025). Most existing studies show that nature immersion in virtual reality can effectively reduce acute experimental or clinical pain (Hughes et al. 2019; Mehesz et al. 2021). However, NOMP is characterised by distinct neurophysiological (e.g., nociception) (Graven-Nielsen 2006; Mense 2003; O’Connor and Cook 1999) and subsequent experiences to experimental and clinical pain types (O’Malley et al. 2024). As no study has assessed whether virtual reality interventions have the same effect on NOMP, we should be cautious to assume that hypoalgesia effects observed in other pain models are generalisable to NOMP.

Thus, the purpose of this study was to assess whether nature scenes presented in virtual reality reduce the intensity of NOMP during motor tasks. Subsequently, the study aimed to assess whether nature immersion through virtual reality enhanced task outputs (e.g., time to exhaustion, force production) compared to sham and control conditions due to potential hypoalgesia. These aims were addressed in two related studies. In Study 1, participants completed a stepped incremental cycling task to exhaustion where their task outputs (power output) were prescribed. In Study 2, participants completed an intermittent isometric handgrip task at a constant perceived effort intensity where they could self-regulate their task outputs (force production). Across both studies, participants performed the tasks under three conditions: with immersive virtual reality projecting a 360˚ nature scene (experimental); a two-dimensional version of the nature scene (sham); and no nature scene (control).

We hypothesised that time to pain onset and task exhaustion would be later in the experimental than the sham and control conditions and that per task iso-time (e.g., 25, 50, 75%), pain intensity and perceived effort would be lower in the experimental than the sham and control conditions (Study 1). We hypothesised that force output would be higher in the experimental versus the sham and control conditions (Study 2). We explored whether task intensity, participant sex, and presence ratings impacted the interactions between NOMP and force production.

## Study 1: materials and methods

This study was registered during data collection at https://osf.io/n92b5. At the time of registration, 10 participants had completed the protocols. The lead researcher in charge of data analysis remained blinded to any participant data until all had completed the protocols and data was fully anonymised. All data collection took place at the corresponding author’s institution between January to April 2025.

### Participants

A total of 12 healthy, active participants volunteered for this study (mean ± SD age: 20.8 ± 0.8 years; 50% each sex). Further participant details can be found in Table S1. Participants had no contraindications to physical exercise and were free from existing pain and any chronic conditions. Before each visit, participants were asked to abstain from food (two hours), caffeine (four hours), vigorous physical exercise (24 h), alcohol (24 h), analgesics (48 h), and to repeat their eating and drinking habits prior to each laboratory visits. Participants were not compensated for their participation. Ethical approval was obtained from the institutional ethics committee (Ref: #8396365) and all participants provided written informed consent prior to completing any data collection sessions. All protocols took place in accordance with the Declaration of Helsinki.

### Sample size calculation and data removal

**Fig. 1:**
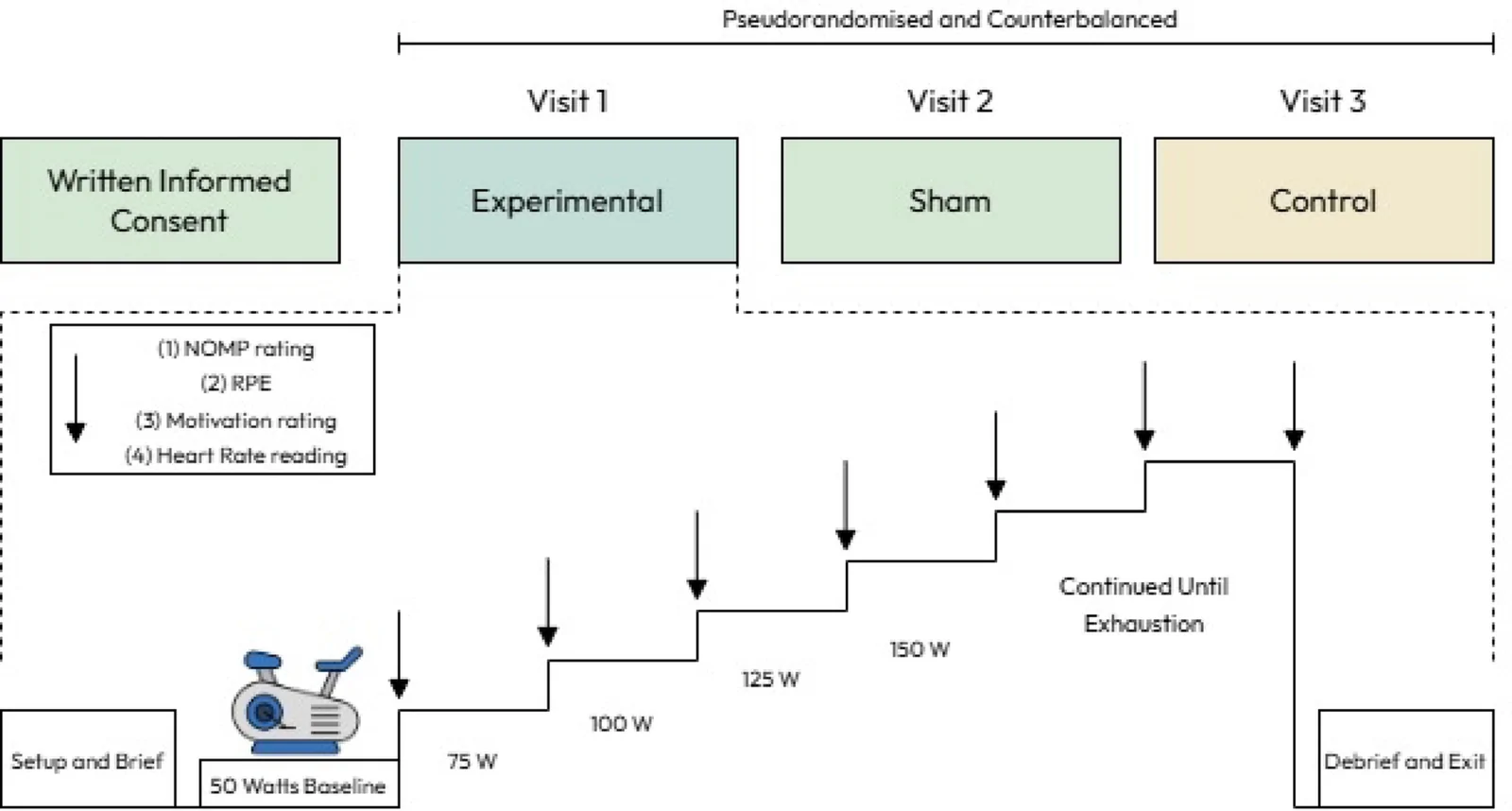
Visual schematic of Study 1 protocols with an inbuilt legend. Abbreviations: NOMP – naturally occurring muscle pain; RPE – ratings of perceived effort; W – Watts

An a-priori power calculation (version 3.1: G*Power, Germany) for within-participant comparisons with a converted effect size of * f* = 0.33 (Mallari et al. 2019), with one group, three measures, α = 0.05, and moderate power of β = 0.80 showed a required sample size of 17 to detect meaningful effects of virtual reality on time to pain onset and task exhaustion with an actual power of β = 0.82. Originally, 18 participants were recruited and completed the study. Data collection was completed by two trainee researchers. After data were collected, the lead researcher and two trainees convened and identified that the data collected by one researcher (*n* = 6) had not been collected according to the pre-registered protocol (Fig. 1). For instance, there were missing data at the point of task exhaustion which subsequently affected iso-time calculations (see analysis). For this reason, data from all conditions from these six participants were removed by the lead research without any data analysis having taken place. A supplementary file can be found on the project page, which shows the excluded data.

### Design

This study used a pseudo-randomised, crossover, repeated measures design. Participants attended three sessions within the same laboratory which had a controlled ambient environment (temperature, humidity, noise) for all sessions. Laboratory visits were spaced two to seven days apart and scheduled at similar times of day (± one hour). Participants completed the same incremental cycling to exhaustion protocol (see Protocol) across each visit (Fig. 1). In each visit, the incremental cycling to exhaustion protocol was completed under one of three conditions: (1) experimental – participants were shown nature scenes within a mounted virtual reality headset (Meta Quest 2: Menlo Park, USA); (2) sham – participants were shown the same nature scenes as within the experimental condition but in two-dimensional form on a screen directly in front of them; and (3) control – no intervention/nature scenes. The nature scenes were 4k footage of the Trail of the 10 Falls (https://youtu.be/HvsBCN34dKY), a subtropical feature of rivers and waterfalls. In the experimental condition, the scenes were projected via two diamond Pentile OLED displays (1,440 × 1,600 resolution; 72 Hz refresh rate) and audio input via two headphones. An inbuilt tracking system provided information about the participants head movements, which adjusted the display environment accordingly so that participants could look around the scene(s) but not control their environment. In the sham condition, participants could only view and not interact with the scene. Therefore, across the experimental and sham conditions, the visual display and audio inputs were the same except for how the individuals could interact with that scene with/without observing what was occurring in the room when protocols were underway. The scene(s) began playing at the start of the participants’ five-minute warm-up. The order of conditions was pseudo-randomised and counterbalanced between visits (see Table S1). There were six possible orders, therefore, each order was completed by two participants each.

### Protocol

After completion of forms and an explanation of the study and scales, participants were fitted with a Polar H10 (Polar: London, UK) heart rate and mounted an Excalibur 3 (Lode bV: Groningen, The Netherlands) cycle ergometer. The heart rate monitor was synchronised with MetaSoft Studio software (Cortex Biophysik GmbH: Leipzig, Germany) to show a beat-by-beat analysis of heart rate (beats·min^− 1^). The resistance of the ergometer was controlled from this software with a preset protocol.

The protocol involved a five-minute constant power (50 Watts) warm-up. Participants were asked to maintain a constant cadence between 80 and 90 revolutions·min^− 1^ throughout the warm-up and incremental cycling task. Immediately following the warm-up, the ramped incremental cycling task began. The task was programmed in a stepped fashion. There was a 25 W stepped increment after each two-minute stage. These steps continued until participants reached exhaustion. The point of exhaustion was demarcated as the point a participant believed they have reached their maximum and stopped cycling or if their cadence dropped below 60 revolutions.min^− 1^ for more than five seconds despite strong verbal encouragement from the researcher. Ratings of naturally occurring muscle pain, perceived effort, and task motivation (see Measures) were rated verbally at the start of the first stage and then the end of each stage including the end of the warm-up and the point of exhaustion if this occurred mid-stage. Average heart rate from a 10 s window (± 5 s from the end of each stage) was recorded. At task exhaustion, the time to task exhaustion (seconds) was recorded.

### Measures

#### Naturally occurring muscle pain

Naturally occurring muscle pain ratings were recorded using the category-ratio (CR)-100 scale (Borg and Borg 2002). Pain was defined to the participants as “the degree of hurt you perceive” (Hawker et al. 2011). Naturally occurring muscle pain focused on the degree of hurt perceived in the working musculature (i.e., both legs). Lower and uppermost boundaries were predefined for each pain rating. For instance, the lowermost boundary of 0 ‘nothing at all’ was related to how participants felt during rest without any exercise. The uppermost boundary of 100 ‘maximal’ denoted the maximal tolerable naturally occurring muscle pain level that a participant felt they could deal with. In relation to naturally occurring muscle pain, this would refer to the point where the exercise task elicited an intensity of pain that would reach/exceed the pain tolerance. Participants were always instructed to consider their pain ratings on a moment-by-moment basis.

Participants were also instructed to alert the researcher when they first detected naturally occurring muscle pain within the legs. This was labelled as the participant ‘pain onset’ (seconds) which denoted the first point at which naturally occurring muscle pain reached a rating of ≥ 1 a.u. on the CR100 scale.

#### Perceived effort

The perception of effort was defined as “how hard, heavy, and strenuous the physical task is” (Marcora 2009). Participants rated their perceived effort according to their experience at the moment the question was asked. The lower and uppermost anchors of the CR100 scale were described according to an incremental cycling task (Malleron et al. 2023). The lowermost boundary of 0 ‘nothing at all’ related to when the participant was rested sitting in the saddle but not doing anything to turn the pedals, and the context-related uppermost boundary of 100 ‘maximal’ was related to when the participants considered they were cycling as hard as they could. Numbers above 100 ‘maximal’ are present on the CR100 scale in case participants considered they had gone beyond what they previously thought as possible.

Naturally occurring muscle pain and perceived effort could be rated on the same scale, as far as instructions given to the participant explicitly dissociate both constructs (Halperin and Emanuel 2020; Pageaux 2016). For instance, an individual could complete a very low intensity cycle for a short duration which would cause them to perceive some effort but involve little/no naturally occurring muscle pain due to the low intensity of the exercise (Cook et al. 1997).

#### Task motivation

Task motivation was rated on a single-item scale according to “please rate how important it is for you to achieve your goal”. Ratings ranged from 0 ‘not at all important’ to 20 ‘extremely important’. Prior to beginning the first stepped incremental cycling task, participants were asked to allocate a personal goal. A default goal was set as “to try to remain engaged in the cycling test for as long as possible” unless participants chose to replace this goal with something else.

#### Environmental presence

At the end of each trial, participants rated environmental presence on a single-item scale relating to “how present did you feel in your environment?”. Ratings ranged from 1 ‘not at all’ to 7 ‘very much’. For this reason, the “environment” in the experimental and sham conditions related to the nature-based video, whereas in the control condition, this related to the room where testing took place.

#### Data processing and analysis

Time to task exhaustion and pain onset did not require any transformation before statistical analysis. Ratings for naturally occurring muscle pain, perceived effort, heart rate, and task motivation were transformed according to 25, 50, 75, and 100% iso-time. Due to the almost linear relationship between both perceived effort and heart rate with task demand (Richter et al. 2008), a linear regression model was applied $\:\left(y=mx+c\right)$. Time (seconds) for each percentage of the total trial was calculated. In Microsoft Excel (Seattle, USA), the obtained perceived effort, heart rate, and task motivation (* y* axis) were then plotted over time (* x* axis) and the linear regression equation was shown. Using the times corresponding to each iso-time percentage as * x* within the equation, the perceived effort, heart rate, and task motivation were calculated for each trial from each participant. The same process was used for naturally occurring muscle pain except that a second-order polynomial regression equation $\:(y = \beta \:_{0} + \beta \:_{1} x + \beta \:_{2} x^{2} + \varepsilon )$ was used to match the sinusoidal trend that naturally occurring muscle pain shows in relation to physical task demand (Cook et al. 1997).

### Statistical analysis

All dependent variable data was exported into JASP (version 0.19: Amsterdam, The Netherlands) and JAMOVI (version 2.31: Sydney, Australia). After checks for normality with a Shapiro-Wilk test, dependent variables were entered into a repeated measures ANOVA or non-parametric equivalent (Friedman’s test) with a single factor to test for condition effects. Greenhouse-Geisser corrections were applied to data that violated sphericity. All main effects were assessed at an alpha level of * p* ≤ 0.05 (two tailed). Main and interaction effects were reported with * F* values, partial eta squared ($\:{\eta\:}_{p}^{2}$) and lower and upper 95% confidence interval (CI) boundaries. Any significant main condition effects were followed with post-hoc Bonferroni comparisons. Post-hoc pairwise comparisons were reported with * t* values, Cohen’s * d*, lower and upper 95% CIs. Pairwise comparisons are reported with the virtual reality condition as the comparator (experimental – sham or experimental – control) or the sham as the comparator (sham – control).

## Study 1: results

According to the data removed from six participants, a post-hoc sensitivity analysis showed that a sample size of 12 participants could detect large effect sizes of * f*(U) = 0.710 (equivalent to $\:{\eta\:}_{p}^{2}$ = 0.156) with a critical value of * F* = 3.443, a statistical power of β = 0.80, ⍺ = 0.05, for within-participants comparisons with three measurements, and with the “as SPSS” option selected. Therefore, findings are interpreted according to these critical values. Additionally, following Schönbrodt and Wagenmakers (2018), we assessed evidential sensitivity post-hoc by examining whether the primary model comparison analyses yielded conclusive Bayes factors ($\:{BF}_{10}$ > 3 or < 0.333; Kass and Raftery 1995).

**Fig. 2:**
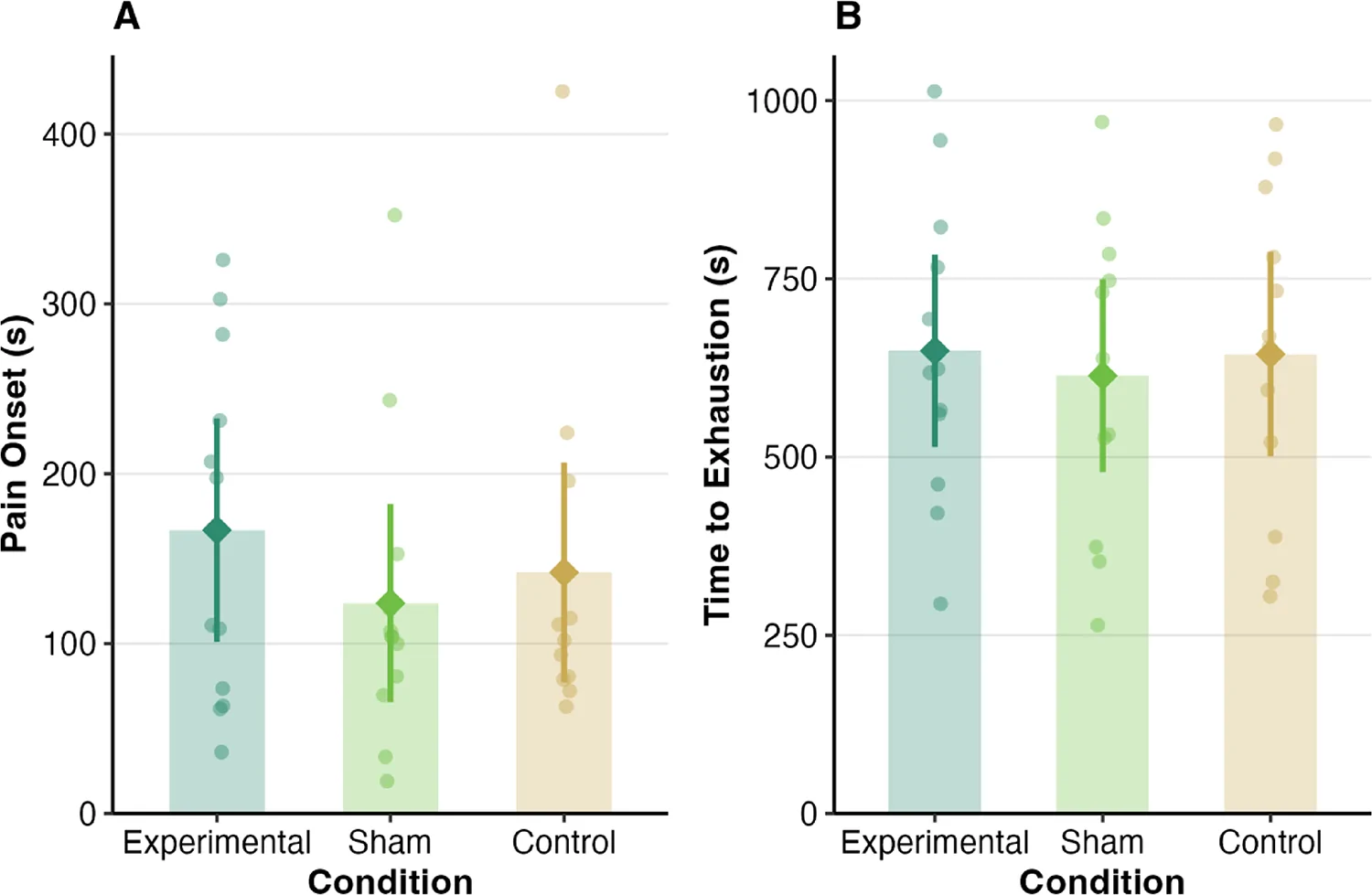
Comparison of naturally occurring muscle pain onset (**A**) and time to task exhaustion (**B**) between conditions. Individual circular plots represent individual data and bars with diamond plot represent group mean. Error bars represent 95% confidence intervals

There was no main effect of condition on pain onset $\:({F}_{\mathrm{2,22}}=1.999,p=.159,\:{\eta\:}_{p}^{2}=0.154)$ though pain onset was later in the experimental condition than the sham and control conditions (Fig. 2A). A Bayes factor indicated weak support of the null hypothesis $\:({BF}_{10}=0.658)$. This means the null hypothesis was 1.520 more likely than the experimental hypothesis. There was no main effect of condition on time to task exhaustion $\:({F}_{\mathrm{2,22}}=1.711,p=.204,\:{\eta\:}_{p}^{2}=0.135)$ though time to exhaustion was longest in the experimental condition (Fig. 2B). A Bayes factor indicated weak support of the null hypothesis $\:({BF}_{10}=0.569)$. This means the null hypothesis was 1.757 more likely than the experimental hypothesis.

**Fig. 3:**
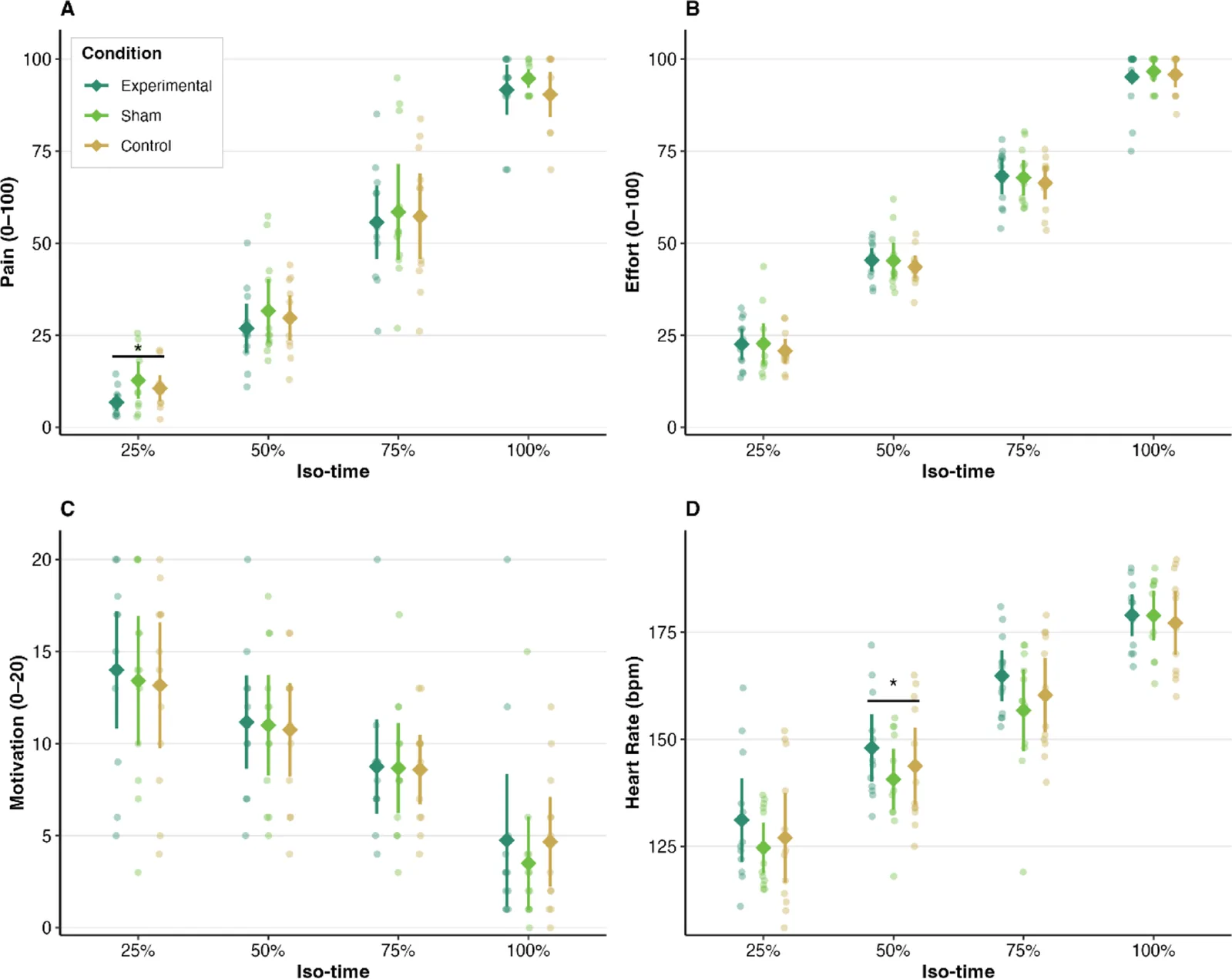
Comparison of naturally occurring muscle pain ratings (**A**), perceived effort ratings (**B**) motivation ratings (**C**) and heart rate (**D**) per 25, 50, 75, and 100% iso-time of the trials between conditions. Individual circular plots represent individual data and bars with diamond plot represent group mean. Error bars represent 95% confidence intervals. Significant main condition effects represented with a = *p* < .05

There was a main effect of condition on NOMP ratings at 25% iso-time of the trial (*F*_2,22_ = 4.518, *p* = .023, $\eta _{p}^{2}$ = 0.291[0.002, 0.500]) . Bayes factor analysis indicated moderate support of the experimental hypothesis $\:({BF}_{10}=3.711).$ Pairwise comparisons showed no differences between the experimental and sham (*t*_11_ = 2.768,* p* = .055,* d* = –1005[–2.195, 0.184]) or control (*t*_11_ = – 2.173,* p* = .158,* d* = – 0.641[– 1.557, 0.276]) conditions though effect sizes were moderate to large (Fig. 3A). Pain ratings at 25% iso-time of the trial were similar between the sham and control conditions $\:({t}_{11}=1.031,p=.973,\:d=0.365[-\mathrm{0.657,1.387}\left]\right)$. There was no main effect of condition on NOMP ratings at 50%, 75%, and 100% iso-time of the trials $\:({p}^{{\prime\:}}s<.369)$. A Bayes factor indicated weak support of the null hypothesis at 50% $\:({BF}_{10}=0.376)$ and 100% $\:({BF}_{10}=0.554)$ iso-time of the trial. Bayes factor analysis indicated moderate support of the null hypothesis at 75% iso-time of the trial $\:({BF}_{10}=0.208)$.

There were no main effects of condition on perceived effort $\:({p}^{{\prime\:}}s>.215)$ and task motivation $\:({p}^{{\prime\:}}s>.717)$ ratings at 25 to 100% iso-time of the trials (Fig. 3B-C). Bayes factors indicated strong support of the null hypothesis for effort ratings at 25 to 100% iso-time of the trial $\:({BF}_{10}>0.272).$ There was a main effect of condition on heart rate at 50% iso-time of the trial $\:({F}_{\mathrm{2,22}}=3.475,p=.049,\:{\eta\:}_{p}^{2}=0.240).$ Bayes factors indicated weak support of the experimental hypothesis $\:\left({BF}_{10}=1.567\right)$ over the null hypothesis. Pairwise comparisons showed that heart rate in the experimental condition was higher than the sham and control conditions but there were no significant differences $\:({p}^{{\prime\:}}s>.078)$ (Fig. 3D). There were no main effects of condition on heart rate at 25, 75, and 100% iso-time of the trial $\:(p{\prime\:}s>.058).$ Bayes factors showed weak support of the null hypothesis for heart rate at 25% iso-time of the trial $\:({BF}_{10}=0.661).$ Bayes factors showed strong support of the null hypothesis for heart rate at 100% iso-time of the trial $\:({BF}_{10}=0.245).$ However, heart rate was higher in the experimental versus other conditions at 75% iso-time of the trial. Though this was statistically insignificant there was a large effect size (*F*_2,22_= 3.246, *p*=.058, $\:{\eta\:}_{p}^{2}$ = 0.228). Bayes factors indicated that the experimental hypothesis was more likely than the null hypothesis for heart rate at 75% iso-time of the trial albeit with weak/inconclusive support $\:({BF}_{10}=1.386).$

There was a main effect of condition on environmental presence ratings (*F*_1.280,14.078_ = 12.081, *p* = .002,$\:{\eta\:}_{p}^{2}$ = 0.523). Pairwise comparisons showed a higher rating of presence in the experimental versus the sham condition (*t*_11_ = 2.966,* p* = .038,* d* = 1.381[-0.172,2.935]) and control versus sham condition (t_11_ = -9.324, *p* <.001,*d* = 2.015[-3.371,-0.659]). Environmental presence was similar between experimental and control conditions (t_11_ = -1.233,* p* = .729,* d* = -0.633[-2.130,0.864]) (Figure S1).

## Study 2: materials and methods

This study was pre-registered at https://osf.io/n92b5 before any data collection took place. All data collection took place at the corresponding author’s institution between January to July 2025.

### Participants

A total of 24 healthy, active participants volunteered for this study (mean ± SD age: 27.0 ± 4.6 years; 50% each sex). Further participant details can be found in Table S2. Participants had no contraindications to physical exercise and were free from existing pain and known chronic conditions. Before each visit, participants were asked to abstain from food (two hours), caffeine (four hours), vigorous physical exercise (24 h), alcohol (24 h), analgesics (48 h), and to repeat their eating and drinking habits prior to both laboratory visits. Participants were not compensated for their participation. Ethical approval was obtained from the institutional ethics committee (Ref: #8396365) and all participants provided written informed consent prior to completing any data collection sessions. All protocols took place in accordance with the Declaration of Helsinki.

### Sample size calculation

Two a-priori calculations were performed in G*Power software (version 3.1, G*Power: Germany). In the first, a within-factors repeated measures ANOVA using a converted effect size from Mallari et al. (2019) of * f* = 0.33, one group, three measures (each condition), α = 0.05, and a moderate power of β = 0.80 showed a required sample size of 17 to detect meaningful effects of virtual reality on pain ratings and force outputs with an actual power of β = 0.82. A second calculation, assessing a within-between interaction for a repeated measures ANOVA with the same converted effect size (* f* = 0.33), two groups (light and hard perceived effort), three measures (each condition), α = 0.05, and a moderate power of β = 0.80 showed a required sample size of 18 to detect meaningful effect of the intensity of perceived effort task on virtual reality effectiveness with an actual power of β = 0.84. Twenty-four participants were recruited to satisfy full pseudo-randomisation and counterbalancing orders (Table S2).

### Design

**Fig. 4:**
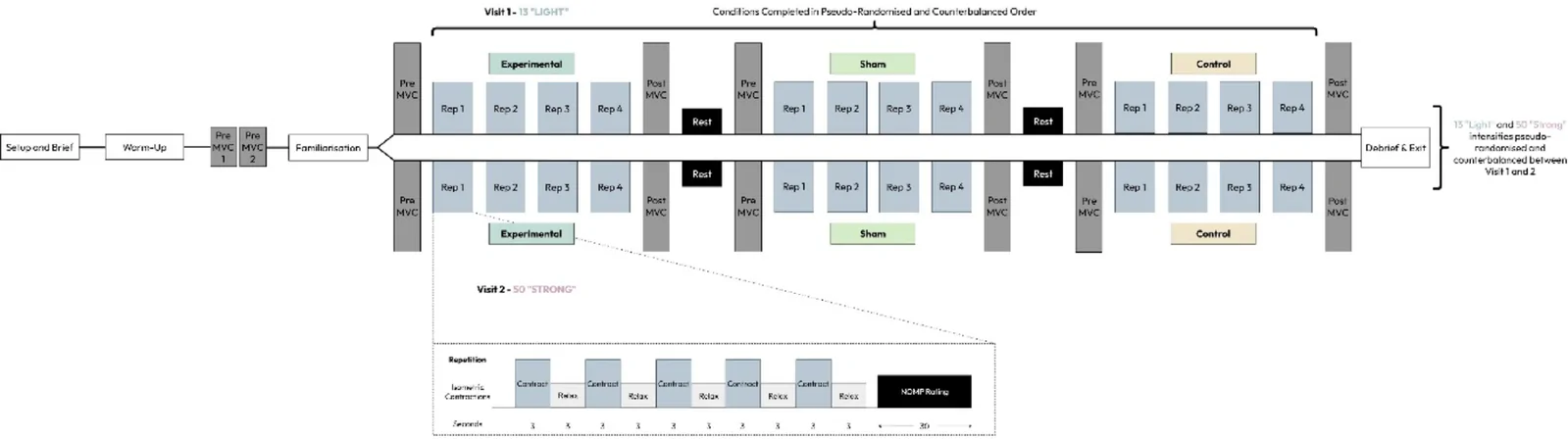
Visual schematic of Study 2 protocols with an inbuilt legend. MVC – maximal voluntary contraction; NOMP – naturally occurring muscle pain; RPE – ratings of perceived effort; W – Watts

This study used a pseudo-randomised, crossover, repeated measures design. Participants attended two sessions within the same laboratory which had a controlled ambient environment. Each visit was separated by two to seven days and scheduled at a similar time of day (± one hour). This study involved intermittent isometric contractions using a handheld device (ADInstruments: Auckland, New Zealand). Participants completed the same warm-up, effort calibrations, familiarisation, and main fixed perceived effort task in both visits (Fig. 4, see Protocol). For each visit, participants were asked to complete the fixed perceived effort task at a light (13/100) or strong (50/100) intensity for each handgrip contraction. Within each visit, the fixed perceived effort handgrip tasks were performed under the same three condition as Study 1. The scene(s) began playing before participants completed their pre-trial maximal voluntary contraction (MVC) for each condition (see Protocol). The order of fixed perceived effort intensities (between visits) and experimental conditions (within visits) was pseudo-randomised and counterbalanced (see Table S2).

### Protocol

#### Handgrip task

Throughout all visits, participants sat in an adjustable chair facing a monitor positioned approximately 70 cm in front of them at eye-level. Participants sat upright with their back resting on the chair support and forearms resting in a supinated position on the table in front of them with elbows at approximately 90–100° flexion. The handgrip (MLT004/ST, ADInstruments: Auckland, New Zealand) was placed in the dominant hand. For all handgrip contractions, participants were asked to squeeze the handgrip without changing the angle of their wrist, elbow, shoulder, or hips (i.e., leaning into the contraction). No feedback of the force was provided to the participants except during maximal voluntary contractions (MVC) and familiarisations. The handgrip dynamometer was connected to a PowerLab 16/35 system (ADInstruments: Auckland, New Zealand). The force signal was digitised online at a sampling frequency of 2 kHz using a computer and stored for analysis with commercially available software (LabChart8 Pro software, ADInstruments: Auckland, New Zealand).

An intermittent structure for the handgrip task was chosen to avoid mechanical compression during a sustained isometric contraction, which inevitably limits the local blood flow even for contractions as low as 20% of MVC peak torque (Lind 2011). Such mechanical compression would create excessive ischaemia that would exacerbate naturally occurring muscle pain due to accumulation of metabolites in the muscle (Aboodarda et al. 2020).

Participants performed the handgrip task according to a fixed light (13) or strong (50) perceived effort intensity on the CR100 scale (Borg and Borg 2002). The start and end of each contraction was signalled to the participants via pre-programmed audio instructions using SuperLab6 software (Cedrus: Santa Barbara, USA). A countdown of 3–2 – 1 was shown on the second monitor in front of the participants before the first audio prompt to contract (“GO”) followed three seconds later by a prompt to relax (“STOP”). During the sham and control condition, participants could see this display, therefore the researcher simply prompted them with “countdown has begun”. During the experimental condition, participants could not see this display, so the researcher prompted the participants by saying the countdown aloud as it was displayed on screen to prime them for the onset of the next repetition. All audio prompts were separated by three seconds until five contractions were performed. After the last “STOP” prompt, there was a 30-second period in which participants rested and provided verbal pain intensity ratings. Participants were instructed to contract and relax in relation to the audio prompts, by reacting as quickly as possible. During each contraction, participants only had the CR100 scale displayed on the second monitor (only visible during the sham and control conditions). No feedback of the force was provided.

#### Procedure

##### Warm up

After completion of forms and an explanation of the study, participants completed a brief warm up for the trial comprising 10 intermittent isometric contractions at what they considered to be 50% of their maximum force. Contractions and rest periods lasted approximately three seconds each and were prompted by the pre-recorded “GO – STOP” audio instructions for the start and end of each contraction. One minute after the warm-up finished, participants were asked to perform a MVC for three seconds. Participants were asked “to squeeze the handgrip as hard as you can for three seconds by going immediately to maximum and holding that as best you can”. Participants performed another MVC one minute after the first. If there was a > ± 10% difference between MVC peak force (Newtons), a third MVC was completed. The MVC peak force was noted by the researcher for the effort calibrations.

##### Effort calibrations

Participants then completed a pyramidical effort calibration task. Participants were asked to squeeze the handgrip for 10 s at an intensity which corresponded to 5–15 – 30–60 – 80–60 – 30–15 – 5% of their MVC peak force. Participants were shown real-time force data from the handgrip. Participants were instructed the force trace must remain within a green band (± 2% of the target force percentage) for the entire 10-second period for the trial to be considered valid and complete. After each 10-second contraction, participants were asked to relax and rate their perceived effort at the end of the contraction (see Measures). This was followed by a 30-second rest and the next intensity of contraction. The pyramidal effort calibration task was completed to allow the participant to squeeze the dynamometer at different force levels, thus experiencing various intensities of effort during handgrip contractions.

##### Familiarisation

Participants were then familiarised to the intermittent isometric handgrip task at a fixed light (13) and strong (50) perceived effort. The fixed perceived effort intensity during the familiarisation corresponded to which intensity was to be completed in the main trials for that visit. The familiarisations involved a shortened version of the main experimental condition lasting approximately five minutes. That is participants completed two repetitions of five contractions for the familiarisation. The intensity of the familiarisation corresponded to the intensity that was assigned for that visit. During the 30-second rest after each repetition, participants verbally rated their naturally occurring muscle pain in their dominant arm. Familiarisations were performed under the same conditions as the experimental condition (with a virtual reality headset display).

##### Fixed perceived effort trials

After the familiarisation and a short five-minute rest, participants then completed the main fixed perceived effort tasks. Main tasks followed the same protocol as the familiarisation except participants completed four repetitions of five contractions for each condition. A MVC was performed one minute before the first repetition started and immediately after the rest and rate period of the last repetition. Within the experimental and sham conditions, the nature scenes were displayed from 15 s before the pre-condition MVC to 15 s after the post-condition MVC. Participants were given a three-minute rest period between each condition. After each condition, participants rated their environmental presence from the condition they just completed. Participants left the laboratory after they completed the last condition.

#### Measures

##### Naturally occurring muscle pain

Ratings for naturally occurring muscle pain used the same definition and scale from Study 1. The same upper and lowermost boundaries from Study 1 for the scale were also described. In this study, participants were instructed to consider their pain ratings according to their perceptions from the end of the last contraction they performed.

##### Perceived effort

Rating for perceived effort during the pyramidical effort calibrations protocol used the same definition and scale from Study 1. The lower and uppermost anchors of the CR100 scale were described according to the isometric handgrip task (Malleron et al. 2023). The lowermost boundary of 0 ‘nothing at all’ related to when the participant was rested with the handgrip resting on the palm of the hand but not squeezing, and the context-related uppermost boundary of 100 ‘maximal’ was related to when the participants completed an MVC where they squeezed as hard as they could (i.e., maximal effort).

##### Environmental presence

At the end of each condition, participants rated environmental presence on a single-item scale relating to “how present did you feel in your environment?”. Ratings ranged from 1 ‘not at all’ to 7 ‘very much’. The “environment” in the VR and sham conditions related to the nature-based video, whereas in the control condition, this related to the room where testing took place.

### Data analysis

Data analysis of force was completed with the LabChart 8 Pro software. Force traces were filtered using a low-pass Butterworth filter of 5 Hz prior to data analysis (Maffliuetti et al. 2016). The peak force, mean force, force-time integral, and peak rate of force development (RFD) were extracted for all MVC and intermittent contractions. The peak force and peak RFD values were normalised by the highest peak value obtained during the MVCs completed before each fixed perceived effort task, then expressed as a percentage. Mean force and force-time integral data were not normalised due to varying contraction durations.

Contraction onset and offset were automatically detected with the Peak Analysis module of LabChart8 Pro, using a 2SD change in raw force threshold, from a two second resting baseline before/after each contraction. Contraction duration (seconds) was also extracted from the LabChart 8 Pro software alongside force data.

Naturally occurring muscle pain ratings were recorded and processed within Microsoft Excel. All participants’ data were averaged according to the: intensity (light versus strong); condition (experimental versus sham versus control); and repetition (repetition 1 versus 2 versus 3 versus 4), thus creating 24 columns of data relating to each of these factors (e.g., peak force for light, experimental, repetition 1). Force measures from MVCs were taken from the MVC with the highest peak force at both pre- and post-condition timepoints. An objective measure of fatigue was calculated by assessing the change in peak force between the highest pre-condition MVC to the highest post-condition MVC for each intensity and condition.

### A-priori statistical analysis

Averaged and compiled data were then exported to JASP (version 0.19) and JAMOVI (version 2.3.28) software for subsequent statistical analysis. Repeated measures ANOVAs were performed with the following factors: perceived effort intensity (light vs. strong); condition (experimental versus sham versus control); repetition (repetition 1 vs. 2 vs. 3 vs. 4). Greenhouse-Geisser corrections were applied to data that violated sphericity.

First, confirmatory analyses determined whether participants understood the instructions of the task. Muscle pain ratings and force production measures were compared between intensities, and contraction duration was compared between intensities, conditions, and repetitions. Consistent with our main aims and pre-registered hypotheses, we focused on the condition effects for muscle pain ratings and force production measures. All main and interaction effects were assessed at an alpha level of * p* ≤ 0.05 (two tailed). Main effects were reported with * F* values, partial eta squared ($\:{\eta\:}_{p}^{2}$) and lower and upper 95% CI boundaries. Post-hoc pairwise comparisons were completed in JASP and JAMOVI and reported with * t* values, Cohen’s * d*, lower and upper 95% CIs. Multiple comparisons were adjusted with Bonferroni corrections in JAMOVI.

### Exploratory statistical analysis

We explored the intensity $\:\times\:$ condition interactions (effect of fixed perceived effort intensity on conditions), condition $\:\times\:$ repetition interactions (changes in condition effects over time), and intensity $\:\times\:$ condition $\:\times\:$ repetition interactions (changes in condition effects over time between fixed perceived effort intensities) for muscle pain ratings and force production measures.

To account for the effects of fatigue, we also performed exploratory analysis which assessed the differences in pre-post condition changes in MVC force variables. The first was a basic students’ * t* test to assess the differences between averaged pre- and post-condition MVC peak force changes in the light versus strong intensity. The second was a 2 (light versus strong) $\:\times\:$ 3 (experimental, sham, control) repeated measures ANOVA to assess peak force changes indexing neuromuscular fatigue-related effects according to intensity and condition.

Next, three exploratory linear regression analyses were completed. The first discerned the effect of naturally occurring muscle pain on force production (force-time integral only) with various interactions. Through a three-step model, muscle pain ratings were added as a factor with subsequent addition of intensity and sex factors to control for their relative and combined effects on force production. Additionally, a students’ * t* test or non-parametric equivalent (Mann-Whitney U) was used to assess sex differences of averaged NOMP ratings. The second exploratory linear regression analysis followed the same two steps as the first linear regression analysis but used environmental presence ratings in place of sex as the third factor.

The third linear regression analysis was another three-step model. Muscle pain ratings were added as a factor (model 1) with the addition of task intensity (model 2) and objective fatigue measures (changes in MVC peak force) as a covariates (model 3). This regression could therefore discount whether fatigue confounded the effect of muscle pain on force production.

## Study 2: results

All statistical outputs for main effects and interactions are reported in a supplementary table (Table S3).

### Manipulation checks

**Fig. 5:**
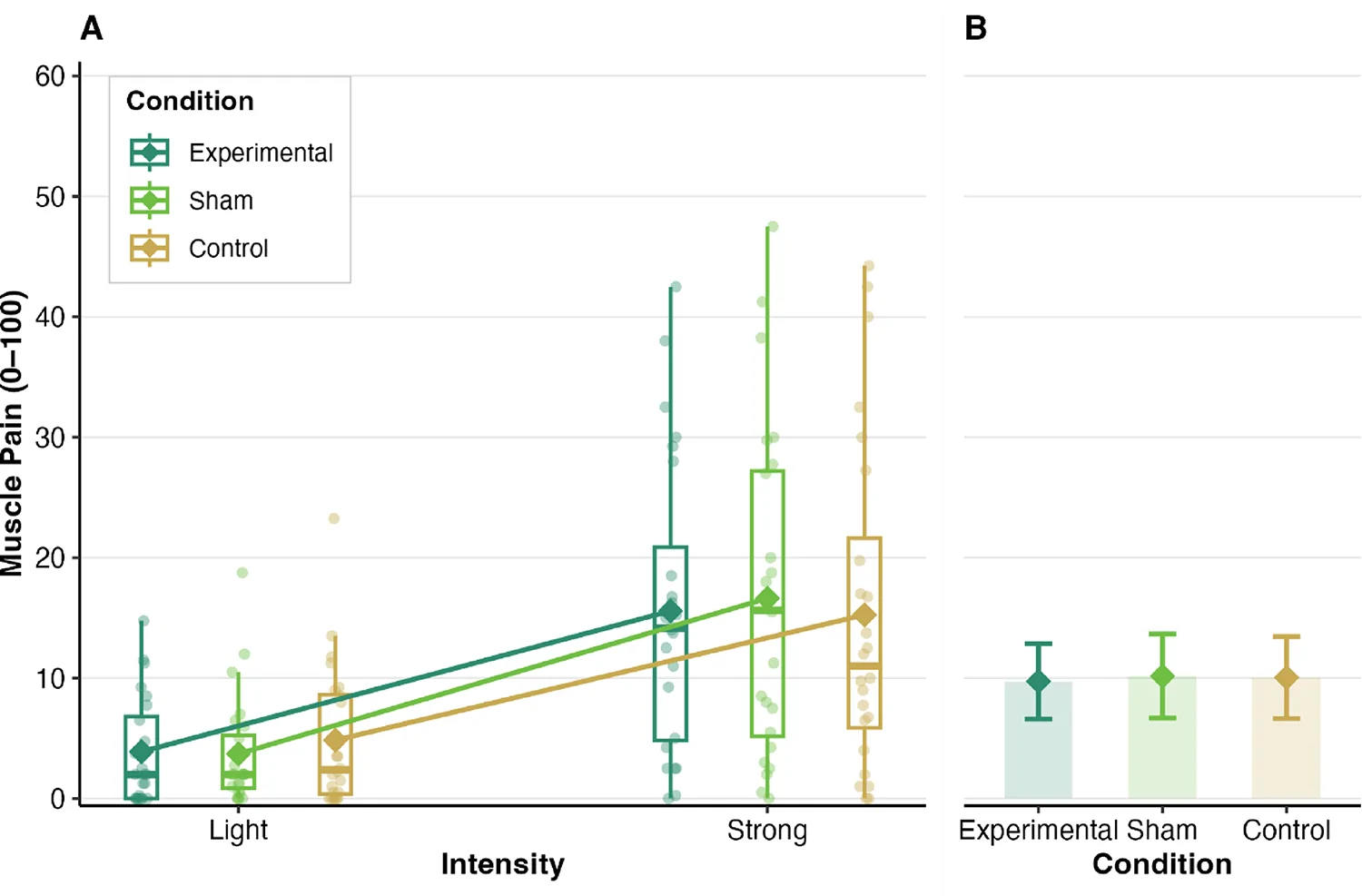
Comparison of naturally occurring muscle pain ratings between conditions and separated by intensity (**A**). Means averaged by condition across both intensities also compared (**B**). Individual circular plots represent individual data and bars with diamond plot represent group mean. Error bars represent 95% confidence intervals

All force parameters were higher within the strong versus the light fixed perceived effort intensity (see Table S3, intensity column). Muscle pain ratings were also higher during the strong versus light intensity (z = -4.197, *p* <.001,*d* =1.000[1.000-1.000]). Mean muscle pain ratings within the light intensity (mean ± SD = 4.2 ± 4.7) corresponded to a “extremely light” description whereas rating within the strong intensity (mean ± SD = 15.8 ± 12.9) corresponded to just above a “light” intensity (Fig. 5). Group mean contraction duration did not differ between the two intensities (*t*_23_ = 0.095,*p* =.925,* d* =0.019[-0.381-0.419]), across conditions (*F*_1.612,37.073_ = 1.789,* p* = .186,$\:{\eta\:}_{p}^{2}$ = 0.072[0.000,0.242]), or between repetitions (*F*_2.071,47.622_ = 1.074,* p* = .352,$\:{\eta\:}_{p}^{2}$ = 0.045[0.000,0.170]) showing participants understood the task instructions and followed the contraction prompts.

### Main condition effects

Ratings of naturally occurring muscle pain were similar across all conditions (*F*_2,46_ = 0.206, * p* =.815, $\:{\eta\:}_{p}^{2}$ = 0.009[0.000,0.083]) each corresponding on average to a rating around 10, just below “light” pain, (Fig. 5).

**Fig. 6:**
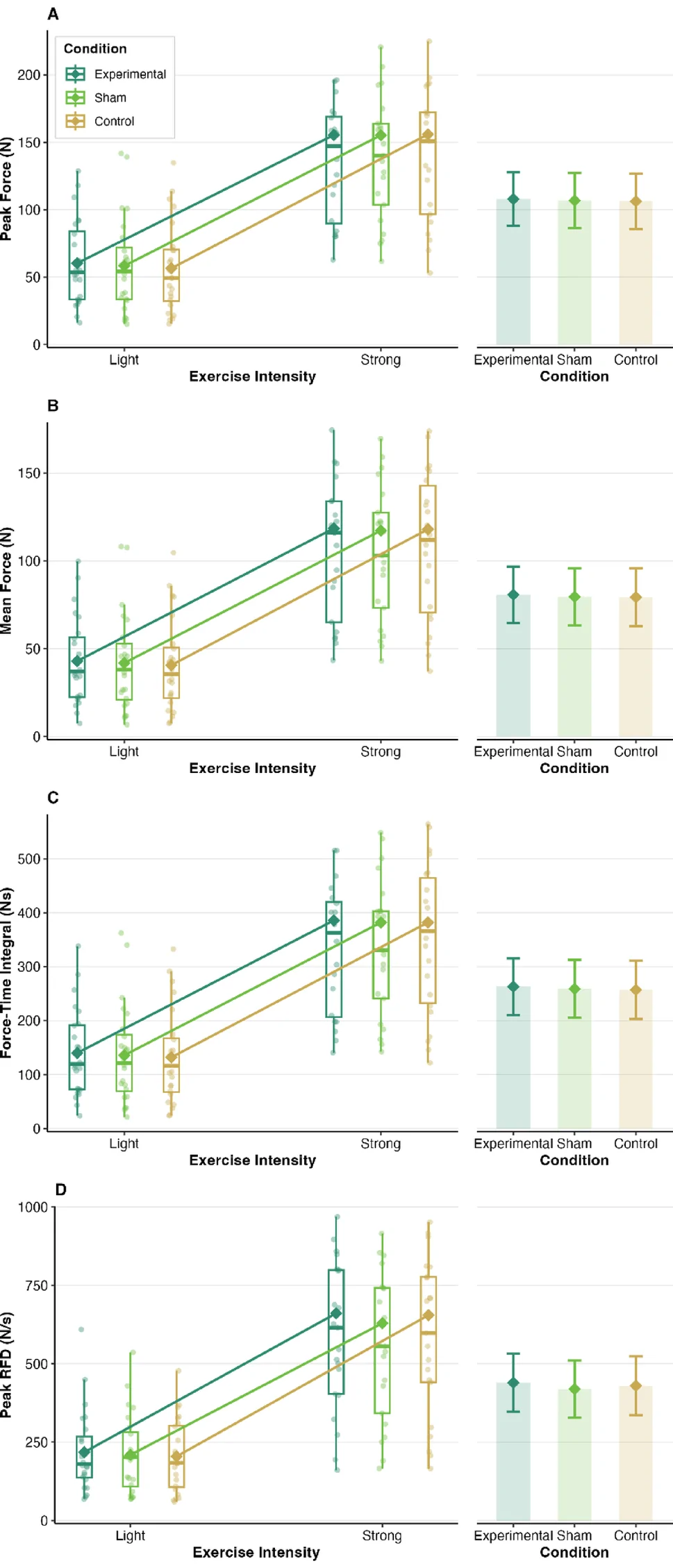
Comparison of peak force (**A**), mean force (**B**), force-time integral (**C**), and peak rate of force development (RFD) (**D**) between conditions. Plots on the left are separated by intensity and means averaged by condition across both intensities presented in plots on the right. Individual circular plots represent individual data and bars with diamond plot represent group mean. Error bars represent 95% confidence intervals

Peak force (*F*_2,46_ = 0.496, * p* = .612,$\:{\eta\:}_{p}^{2}$ = 0.021[0.000,0.124]), mean force (*F*_2,46_ = 0.710, * p* = .497,$\:{\eta\:}_{p}^{2}$= 0.030[0.000,0.145]), and force-time integral (*F*_2,46_= 1.150, * p* = .325, $\:{\eta\:}_{p}^{2}$= 0.048[0.000,0.179]) were almost identical across all conditions (Fig. 6A-C). Peak RFD was highest in the experimental condition and lowest in the sham condition with moderate effects trending near significance (*F*_2,46_ = 2.891, *p* = .066, $\:{\eta\:}_{p}^{2}$ = 0.112[0.000,0.270]) (Fig. 6D). Normalised peak force was similar across all three conditions (*F*_2,46_= 0.223,*p* = .801, $\:{\eta\:}_{p}^{2}$ = 0.010[0.000,0.087]), as was normalised peak RFD (*F*_1.484,34.124_ = 1.369, * p* =.263, $\:{\eta\:}_{p}^{2}$= 0.056[0.000,0.228] (Figure S2).

Exploratory pairwise comparisons (as no repeated measures ANOVAs indicated main condition effects) showed no differences peak force, mean force, force-time integral, peak RFD, normalised peak force, and normalise peak RFD values between condition $\:{(p}^{{\prime\:}}s>.097)$. Muscle pain also showed no differences between conditions $\:{(p}^{{\prime\:}}s\ge\:1.000)$.

### Exploratory analysis - interacting effects

No intensity $\:\times\:$ condition interactions were observed for peak force, mean force, force-time integral, or peak RFD $\:{(p}^{{\prime\:}}s<.456)$ indicating that the fixed perceived effort intensity had no further influence on differences between condition. No intensity $\:\times\:$ condition interactions were observed for normalised peak force or normalised peak RFD $\:({p}^{{\prime\:}}s=.087$ and * .731* respectively* )*. There was an intensity $\:\times\:$ condition interaction for muscle pain ratings (*F*_1.622,37.297_ = 4.656,* p* =.022, $\:{\eta\:}_{p}^{2}$ = 0.168[0.002,0.366] which indicated that whilst mean muscle pain ratings were similar across conditions during the light intensity, mean muscle pain ratings were in fact slightly higher in the experimental to the sham condition, which in turn, was slightly higher than the control condition. Pairwise comparisons of condition effects separated by intensity did not reveal any notable differences in either light $\:({p}^{{\prime\:}}s>1.000)$ or strong intensities $\:({p}^{{\prime\:}}s>1.000)$.

No condition $\:\times\:$ repetition interactions were observed for peak force, mean force, or force-time integral $\:{(p}^{{\prime\:}}s>.356)$ suggesting a similar decline in force production across conditions. There was a condition $\:\times\:$ repetition interaction for peak RFD (*F*_6,138_ = 2.358,* p* =.034, $\:{\eta\:}_{p}^{2}$ = 0.093[0.000,0.157]) Closer inspection of plots showed mean peak RFD minorly increased in the later repetitions of the experimental condition, minorly decreased in the later repetitions of the sham condition and remained relatively constant across repetitions in the control condition.

Normalised peak force showed no condition $\:\times\:$ repetition interaction * (p=.726)* but normalised peak RFD did show an interaction (*F*_6,138_ = 2.824,* p* =.013, $\:{\eta\:}_{p}^{2}$ = 0.109[0.006,0.178]. Inspection of plots showed mean normalised peak RFD remained relatively constant within the experimental condition, minorly increased in later repetitions of the sham condition, and minorly decreased in later repetitions of the control condition. No condition $\:\times\:$ repetition interaction was observed for muscle pain ratings * (p=.148)*.

Exploration of intensity $\:\times\:$ condition $\:\times\:$ repetition interactions showed no significant interactions $\:{(p}^{{\prime\:}}s>.243)$ for any of the raw or normalised force measures. Muscle pain ratings also showed no intensity $\:\times\:$ condition $\:\times\:$ repetition interaction * (p=.365)*. There was an intensity $\:\times\:$ condition $\:\times\:$ repetition interaction for contraction duration $\:({F}_{\mathrm{6,138}}=3.126,p=.007,\:{\eta\:}_{p}^{2}=0.120[\mathrm{0.011,0.191}\left]\right)$.

Further exploratory analyses relating to time-on-task (fatigue) effects and linear regression analyses are provided in Supplementary Materials S3-9.

## Discussion

This two-part study aimed to assess whether nature immersion presented in virtual reality reduces the intensity of naturally occurring muscle pain during motor tasks versus a control or sham condition. We also investigated whether there were any subsequent effects to enhance task outputs (e.g., time to exhaustion, force production) compared to a sham or control condition. Contrary to our a-priori hypotheses, nature immersion through a virtual reality device did not delay naturally occurring muscle pain onset (Study 1), prolong task engagement (Study 1), or increase force production (Study 2) compared to sham or control conditions.

During the incremental cycling task, pain, effort, and heart rate increased with task demand. During the fixed perceived effort task, force production was higher and participants reported more muscle pain when exercising at a strong versus light effort. Contraction times did not differ between intensities or conditions. These results confirm that the experimental manipulations were successful, and the task instructions of both studies were understood.

The majority of our analysis showed null effects. That is the virtual reality intervention which immersed participants in a passive nature environment did not reduce NOMP intensity compared to sham or control conditions and/or improve motor performance outputs. In Study 1, a potential reason for the lack expected differences is due to the incongruence between the intervention and the physical task. Other studies have illustrated that virtual reality interventions which involve immersive environments which update according to the participants actions, and thus, provide relevant visual and vestibular cues can minimise fatigue, breathlessness (Biller et al. 2026) and perceived effort (Bruce et al. 2025). Alternatively, the participants in Study 1 performed an active cycling task whilst observing a passive nature scene, meaning the actions of the participants were not reflected in the virtual environment. This may have contributed to a reduced sense of environmental presence – notably participants reported a lower environmental presence in the experimental condition than the control. Thus, reducing the “distractive” element of virtual reality and its ability to cause hypoalgesia (Hoffman et al. 2004a, b). The sensory conflicts between the virtual environment and the participants’ actions may also have increased cognitive load as they tried to reconcile these differences or increased cybersickness (Maneuvrier et al. 2023) which together could have negated any distractive or motivational effects that virtual reality has shown in previous studies (Gupta et al. 2018; Malloy and Milling 2010). Collectively, these methodological considerations indicate that future research may benefit from using a more “congruent” virtual reality intervention which updates the environment according to the user’s actions to maintain any potential hypoalgesia effects.

Despite mostly null effects, in Study 1 we did observe a slightly later onset of NOMP and lower pain intensity at 25% iso-time of the cycling trial during the virtual reality condition. Therefore, if virtual reality does have any effect, it may be limited to when NOMP is at a light-moderate intensity. Indeed, at stronger pain intensities it is unlikely a nature immersion intervention would be salient enough to effectively alter the central processing of nociceptive signals (Mallari et al. 2019; Medina and Hughes 2025). Furthermore, previous studies have illustrated that effective hypoalgesia from nature immersion in virtual reality often occurs when stimulations elicit light-moderate pain (Mehesz et al. 2021; Steininger et al. 2025). As such, we speculate that virtual reality interventions are potentially limited to pain intensities that are low-moderate. Future research could consider a meta-analytic review of virtual reality interventions on various pain types and pain intensities as an intriguing way to confirm this.

Meanwhile, the lack of main condition effects In Study 2 may be due to the relatively low NOMP across both perceived effort intensities. The NOMP intensity averaged ~ 5/100 in the light and ~ 16/100 in the strong intensity, with a mean NOMP intensity of ~ 10/100 across both intensities which corresponds to between a “very light” to “light” description. Subsequently, this may have limited the potential for the virtual reality intervention to elicit a noticeable hypoalgesia effect. Though we noted earlier that virtual reality interventions may be limited to light-moderate pain intensities (Mehesz et al. 2021; Steininger et al. 2025), these studies utilised a different pain scale which demarcates “moderate” at 50/100, whereas we used the CR100 scale (Borg and Borg 2002) which denotes “moderate” at ~ 25/100. In response, a future study could trial a fixed force/power or fixed pain task design to elicit more controlled intensities of NOMP during a physical task and virtual reality exposures. Alongside which, consistent use of numerical rating or visual analogue scales is highly recommended for consistency between studies.

Next, we also expand on our results in relation to the broader field of pain. Part of the rationale for this study was to examine if nature immersion in virtual reality could reduce NOMP in the same way studies have shown it can effectively reduce experimental pain (e.g., Hoffman et al. 2004a, b; Hughes et al. 2019; Medina and Hughes 2025; Steininger et al. 2025; Mehesz et al. 2021). Virtual reality interventions can effectively attenuate experimental pain modalities by engaging descending inhibitory pathways and reducing the central processing of pain (Medina and Hughes 2025; Steininger et al. 2025). During experimental pressure (e.g., pinprick, cuff algometry), thermal (e.g., cold water immersion), or electrical stimulations, virtual reality interventions are likely to be effective because the subsequent pain is transient and distinct from the ongoing task (Handwerker and Kobal 1993; Medina and Hughes 2025; Morin and Bushnell 1998; Steininger et al. 2025). This means that central pain modulation can take greater effect (Medina and Hughes 2025) but some studies caveat this to conditions when the threat value of the pain is low (Van Damme et al. 2008). On the other hand, NOMP is tightly coupled with task demands (Cook et al. 1997) and the related changes in muscle homeostasis (Mense 2003; Pollak et al. 2014). Therefore, during a physical task, individuals often remain cognisant of their NOMP as a crucial indicator of task performance (Mauger 2014). Considering that NOMP is largely driven by peripheral components (O’Connor and Cook 1999; Mense 2003) and is closely linked to the ongoing task, it is plausible that virtual reality interventions which target the central pain processing mechanisms intensity (Gupta et al. 2018; Mehesz et al. 2021) are less effective at modulating NOMP. To our knowledge, our study is the first to explore whether pain type does impact the effectiveness of virtual reality interventions for pain modulation. We would encourage future studies to re-examine our results with targeted measures of nociceptive processing, descending inhibition, or conditioned pain modulation (CPM) to better elucidate this phenomenon and confirm whether virtual reality interventions are pain-type dependent.

Finally, across our two studies we explored whether the effects of a virtual reality intervention on motor behaviour depend on the motor task type. When completing physical tasks such as an incremental cycling test, participants have limited options to self-regulate their motor behaviour, as the sole requirement of the task is to match a given force/power (O’Malley et al. 2023). While participants can change some aspects of their task performance (e.g., force steadiness, adapted movement patterns) (Arvanitidis et al. 2025; Hodges 2016), overall, participants are dichotomised into either a ‘continue’ or ‘quit’ decision as performance is dictated by the participant meeting or falling short of the prescribed task demand (Marcora 2019). In contrast during a motor task which allowed participants to freely regulate (i.e., increase or decrease) their force, as long as they maintained a constant perceived effort intensity, it may be possible to observe more subtle changes to motor behaviour (O’Malley et al. 2023) with and without a virtual reality intervention. However, NOMP intensity and time to task exhaustion were similar in most conditions in Study 1, and muscle pain intensity and force production were almost identical across all conditions in Study 2. Therefore, our data indicates that nature immersion via a virtual reality intervention has similar null effects on NOMP and task performance across different types of motor task.

### Limitations and future research

Throughout our discussion, we have highlighted that because virtual reality interventions target the descending pain modulation and central processing of pain (Gupta et al. 2018; Medina and Hughes 2025; Medina et al. 2024; Mehesz et al. 2021), a virtual reality intervention may be less effective for pain like NOMP which has a strong peripheral component. Likewise, though the virtual reality condition had a high visual quality, the activity within the virtual reality contrasted with the participants’ movements (e.g., cycling, handgrip). Therefore, this may have created a mismatch between visual and vestibular sensations and physical movement, potentially increasing cognitive demands, reducing participants’ sense of immersion/presence, and thus reducing the effects of virtual reality compared to other studies which have used more compatible virtual reality environments to the task being completed (e.g., Biller et al. 2026; Bruce et al. 2025).

Linked to this, presence is a multifaceted construct (Harris et al. 2020; Slater et al. 2022). Our single-item environmental presence measure was mainly included as a confirmatory check that the virtual reality environment was more “immersive” than the sham version and for exploration on whether these ratings might have impacted the NOMP-force dynamic. Specifically, the phrasing of the question “…in the environment you were in” likely explains why the experimental condition involved lower presence ratings than the control as participants responses related to the immersive environment for the experimental condition but the actual room for the control condition. Together this indicates that our single-item measure is unlikely to fully capture the presence phenomenon, and we would advocate for future studies to incorporate more detailed questionnaires. This would allow researchers to provide more mechanistic accounts about why virtual reality may affect NOMP and motor performance and how presence may play a mediatory role in this relationship (Slater et al. 2022).

Another consideration is that our studies have strictly assessed perceptual (i.e., pain intensity) and behavioural (i.e., power and force) changes during a virtual reality intervention. To better understand the definitive role of central pain modulation, we propose that future studies could benefit from combining perceptual and behavioural measures with neurophysiological assessments like CPM. Through CPM measures, researchers can judge how effectively an individual can modulate pain via central mechanisms (Bannister et al. 2025) during virtual reality exposure. Therefore, if virtual reality exerts its effects primarily through these central mechanisms, individuals with stronger CPM responses (i.e., greater endogenous inhibition) would be expected to show more pronounced reductions in pain (Bannister and Hughes 2023) compared to sham or control conditions, confirming this mechanism.

Lastly, we must acknowledge that a-priori calculations determined a required sample size of 17 participants for Study 1 to detect meaningful moderate effects. Although 18 participants were recruited and completed the protocols, data from six participants was removed during data processing because the lead researcher identified that the trainee researcher had missing data relating to time to task exhaustion measures (main dependent variables) which was also crucial for the calculation of the iso-time values. A sensitivity analysis with 12 participants collected by the other researcher(s) indicated a critical * F* value of 3.443. Notably, the significant, large effect on pain at 25% iso-time exceeded this critical value (* F=4.518*), however, others which showed non-significant but moderate-large effect sizes like time to pain onset (* F=1.999*) and time to task exhaustion (* F=1.711*) were below this critical value but Bayes factors showed weak support for the null hypothesis $\:({BF}_{10}>0.333)$. Therefore, we should acknowledge that Study 1 of this project is likely underpowered to detect small-medium effects, and readers should caution their interpretation of these results. Nevertheless, exploratory data is critical in helping generate a-priori hypotheses for future research (Briand et al. 2025). Furthermore, the combination of both of our related studies helps strengthen the overall body of evidence about the effects of nature immersion exposure in virtual reality on NOMP and motor performance. Further investigations involving equivalence testing or Bayesian analysis could further explore the true effects of virtual reality interventions on NOMP. Considering the ubiquitous nature of muscle pain and the limiting effect it has on motor task performance; we challenge researchers to continue seeking effective non-invasive and non-pharmacological interventions that can attenuate pain and improve performance and health (e.g., exercise uptake and adherence) outcomes.

## Conclusion

In conclusion, most of our results indicate that nature immersion through a virtual reality intervention does not reduce NOMP or enhance motor performance across different task types. We did observe a lower NOMP intensity at 25% iso-time of the trial in Study 1 which hints that any potential hypoalgesia effects from virtual reality interventions on NOMP could be restricted to low–moderate pain intensities when the distracting sensory cue in virtual reality are salient enough to impact the processing of nociceptive signals. In the wider scope of pain research, these findings suggest that the effectiveness of virtual reality for pain modulation could be dependent on pain type and pain intensity. Namely, virtual reality interventions may be less effective for NOMP because of its dominant peripheral component compared to experimental and clinical pain types which are distinct from the ongoing physical task. To better confirm this, we advocate for future research to examine the interaction between central and peripheral mechanisms, incorporating neurophysiological measures to clarify underlying pathways and identify conditions under which virtual reality interventions may be most effective to reduce pain intensity. Doing so, will inform practitioners whether virtual reality interventions are suitable for an individual’s pain according to its type and intensity.

## Supplementary Information

Below is the link to the electronic supplementary material.

Supplementary Material 1

## Data Availability

These studies were preregistered at https://osf.io/n92b5. Anonymised data and supplementary materials can also be found within the project page at https://osf.io/eaqbg/. This study has been preprinted via the OSF project page and PsychArXiv. Any additional information or questions relating to data can be sent to the corresponding/lead author who can provide any further details if required.

## Abbreviations

CI: Confidence interval
CPM: Conditioned pain modulation
CR100: Category-ratio 100
NOMP: Naturally occurring muscle pain
VR: Virtual reality

## Declarations

### AI generative statement

No artificial intelligence generated content was involved in the production of this article.

## References

[CR1] Aboodarda SJ, Iannetta D, Emami N, Varesco G, Murias JM, Millet GY (2020) Effects of pre-induced fatigue vs. concurrent pain on exercise tolerance, neuromuscular performance and corticospinal responses of locomotor muscles. J Physiol 598:285–302. 10.1113/JP278943 31826296

[CR2] Angius L, Pageaux B, Hopker J, Marcora SM, Mauger AR (2016) Transcranial direct current stimulation improves isometric time to exhaustion of the knee extensors. Neurosci 339:363–375. 10.1016/j.neuroscience.2016.10.028 27751960

[CR3] Arvanitidis M, Falla D, Sanderson A, Martinez-Valdes E (2025) Does pain influence control of muscle force? A systematic review and meta-analysis. Eur J Pain 29:e4716. 10.1002/ejp.4716 PMC11671343 39176440

[CR4] Bannister K, Hughes S (2023) One size does not fit all: towards optimising the therapeutic potential of endogenous pain modulatory systems. Pain 164:e5–e9. 10.1097/j.pain.0000000000002697 PMC9756434 35594517

[CR5] Bannister K, Patel R, Hughes S (2025) The descending modulation of pain. Pain 166:S55–S59. 10.1097/j.pain.0000000000003683 41086329

[CR6] Biller L, Starling L, Sinha A, Dearlove D, Bruce R, Runswick O, Taylor S, Sergeant M, Finnegan S, Pattinson K (2026) Incongruent virtual reality attenuates breathlessness and leg fatigue during stationary cycling. Virtual Real 30:141. 10.1007/s10055-026-01391-6 PMC13372920 42465694

[CR7] Borg E, Borg G (2002) A comparison of AME and CR100 for scaling perceived exertion. Acta Psychol 109:157–175. 10.1016/S0001-6918(01)00055-5 11820425

[CR8] Briand J, Mangin T, Tremblay J, Pageaux B (2025) Bridging inductive and deductive reasoning: A proposal to enhance the evaluation and development of models in sports and exercise science. Sports Med 55:2707–2719. 10.1007/s40279-025-02289-0 40751102

[CR9] Bruce RM, Rafferty GF, Finnegan SL, Sergeant M, Pattinson KTS, Runswick OR (2025) Incongruent virtual reality cycling exercise demonstrates a role of perceived effort in cardiovascular control. J Physiol 603:5149–5161. 10.1113/JP287421 PMC12456392 39754534

[CR10] Cook DB, O’Connor PJ, Eubanks SA, Smith JC, Lee M (1997) Naturally occurring muscle pain during exercise: assessment and experimental evidence. Med Sci Sports Exerc 29:999–1012. 10.1097/00005768-199708000-00004 9268956

[CR12] Gendolla GHE, Wright RA, Richter M (2019) Advancing issues in motivation intensity research: updated insights from the cardiovascular system. In: Ryan RM (ed) The Oxford handbook of human motivation, 2nd edn. Oxford University Press, New York, NY, pp 372–392. 10.1093/oxfordhb/9780190666453.013.21.

[CR13] Graven-Nielsen T (2006) Fundamentals of muscle pain, referred pain, and deep tissue hyperalgesia. Scand J Rheumatol 35:1–43. 10.1080/03009740600865980 16997767

[CR14] Gupta A, Scott K, Dukewich M (2018) Innovative technology using virtual reality in the treatment of pain: Does it reduce pain via distraction, or is there more to it? Pain Med 19:151–159. 10.1093/pm/pnx109 29025113

[CR15] Halperin I, Emanuel A (2020) Rating of perceived effort: Methodological concerns and future directions. Sports Med 50:679–687. 10.1007/s40279-019-01229-z 31745731

[CR16] Halperin I, Vigotsky AD (2024) An integrated perspective of effort and perception of effort. Sports Med 54:2019–2032. 10.1007/s40279-024-02055-8 PMC11329614 38909350

[CR17] Handwerker HO, Kobal G (1993) Psychophysiology of experimentally induced pain. Physiol Rev 73:639–671. 10.1152/physrev.1993.73.3.639 8332641

[CR18] Harris DJ, Bird JM, Smart PA, Wilson MR, Vine SJ (2020) A framework for the testing and validation of simulated environments in experimentation and training. Front Psychol 11:605. 10.3389/fpsyg.2020.00605 PMC7136518 32296379

[CR19] Hawker GA, Mian S, Kendzerska T, French M (2011) Measures of adult pain: Visual Analog Scale for Pain (VAS Pain), Numeric Rating Scale for Pain (NRS Pain), McGill Pain Questionnaire (MPQ), Short-Form McGill Pain Questionnaire (SF-MPQ), Chronic Pain Grade Scale (CPGS), Short Form-36 Bodily Pain Scale (SF-36 BPS), and Measure of Intermittent and Constant Osteoarthritis Pain (ICOAP). Arthritis Care Res 63:S240–S252. 10.1002/acr.20543 22588748

[CR20] Hodges PW (2016) Motor control and pain. In: Sluka KA (ed) Mechanisms and management of pain for the physical therapist. Wolters Kluwer Health Adis, Philadelphia, PA, pp 67–82

[CR21] Hofbauer RK, Rainville P, Duncan GH, Bushnell MC (2001) Cortical representation of the sensory dimension of pain. J Neurophysiol 86:402–411. 10.1152/jn.2001.86.1.40 11431520

[CR22] Hoffman HG, Richards TL, Coda B, Bills AR, Blough D, Richards AL, Sharar SR (2004a) Modulation of thermal pain-related brain activity with virtual reality: evidence from fMRI. NeuroReport 15:1245–1248. 10.1097/01.wnr.0000127826.73576.91 15167542

[CR23] Hoffman HG, Sharar SR, Coda B, Everett JJ, Ciol M, Richards T, Patterson DR (2004b) Manipulating presence influences the magnitude of virtual reality analgesia. Pain 111:162–168. 10.1016/j.pain.2004.06.013 15327820

[CR24] Hughes SW, Zhao H, Auvinet E, Strutton PH (2019) Attenuation of capsaicin-induced ongoing pain and secondary hyperalgesia during exposure to an immersive virtual reality environment. Pain Rep 4:e790. 10.1097/PR9.0000000000000790 PMC6903343 31984295

[CR25] Kass RE, Raftery AE (1995) Bayes factors. J Am Stat Assoc 90:773–795

[CR26] Lind AR (2011) Cardiovascular adjustments to isometric contractions: static effort. In: Shepherd JT, Abboud FM (eds) Handbook of physiology. The cardiovascular system. peripheral circulation and organ blood flow. American Physiological Society, Bethesda, MD, pp 329–370

[CR27] Maffliuetti NA, Aagaard P, Blazevich AJ, Folland J, Tillin N, Duchateau J (2016) Rate of force development: Physiological and methodological considerations. Eur J Appl Physiol 116:1091–1116. 10.1007/s00421-016-3346-6 PMC4875063 26941023

[CR28] Mallari B, Spaeth EK, Goh H, Boyd BS (2019) Virtual reality as an analgesic for acute and chronic pain in adults: a systematic review and meta-analysis. J Pain Res 12:2053–2085. 10.2147/JPR.S200498 PMC6613199 31308733

[CR29] Malleron T, Har-Nir I, Vigotsky AD, Halperin I (2023) Rating of perceived effort but relative to what? A comparison between imposed and self-selected anchors. Psychol Sport Exerc 66:102396. 10.1016/j.psychsport.2023.102396 37665858

[CR30] Malloy KM, Milling LS (2010) The effectiveness of virtual reality distraction for pain reduction: A systematic review. Clin Psychol Rev 30:1011–1018. 10.1016/j.cpr.2010.07.001 20691523

[CR31] Maneuvrier A, Ceyte H, Renaud P, Morello R, Fleury P, Decker LM (2023) Virtual reality and neuropsychological assessment: An analysis of human factors influencing performance and perceived mental effort. Virtual Real 27:849–861. 10.1007/s10055-022-00698-4

[CR32] Marcora SM (2009) Perception of effort. In: Goldstein EB (ed) Encyclopedia of Perception. Sage, Thousand Oaks, CA, pp 677–680

[CR33] Marcora SM (2019) Psychobiology of fatigue during endurance exercise. In: Meijen C (ed) Endurance performance in sport, 1st edn. Routledge, London, UK, pp 15–35. 10.4324/9781315167312.

[CR34] Mauger AR (2014) Factors affecting the regulation of pacing: current perspectives. Open Access J Sports Med 5:209–214. 10.2147/OAJSM.S38599 PMC4161528 25228823

[CR36] Medina S, Hughes SW (2025) Immersion in nature through virtual reality attenuates the development and spread of mechanical secondary hyperalgesia: a role for insulo-thalamic effective connectivity. Pain 166:2181–2193. 10.1097/j.pain.0000000000003701 40717606

[CR35] Medina S, Clarke S, Hughes SW (2024) Virtual reality-based analgesia: towards a novel framework for the biopsychosocial management of chronic pain. Br J Anaesth 133:486–490. 10.1016/j.bja.2024.06.005 38997839

[CR37] Mehesz E, Karoui H, Strutton PH, Hughes SW (2021) Exposure to an immersive virtual reality environment can modulate perceptual correlates of endogenous analgesia and central sensitization in healthy volunteers. J Pain 22:707–714. 10.1016/j.jpain.2020.12.007 33465506

[CR38] Mense S (2003) The pathogenesis of muscle pain. Curr Pain Headache Rep 7:241–247. 10.1007/s11916-001-0023-6 14604500

[CR39] Morin C, Bushnell CM (1998) Temporal and qualitative properties of cold pain and heat pain: a psychophysical study. Pain 74:67–73. 10.1016/S0304-3959(97)00152-8 9514562

[CR40] Norbury R, Smith SA, Burnley M, Judge M, Mauger AR (2022) The effect of elevated muscle pain on neuromuscular fatigue during exercise. Eur J Appl Physiol 122:113–126. 10.1007/s00421-021-04814-1 PMC8748369 34586471

[CR42] O’Malley CA, Fullerton CL, Mauger AR (2023) Test-retest reliability of a 30-min fixed perceived effort cycling exercise. Eur J Appl Physiol 123:721–735. 10.1007/s00421-022-05094-z PMC10030391 36436029

[CR43] O’Malley CA, Smith SA, Mauger AR, Norbury R (2024) Exercise-induced pain within endurance exercise settings: Definitions, measurement, mechanisms and potential interventions. Exp Physiol 109:1446–1460. 10.1113/EP091687 PMC11363130 38985528

[CR41] O’Connor PJ, Cook DB (1999) The neurobiology, measurement, and laboratory study of pain in relation to exercise in humans. Exerc Sport Sci Rev 27:267–292 10791016

[CR44] Pageaux B (2016) Perception of effort in Exercise Science: Definition, measurement and perspectives. Eur J Sport Sci 16:885–894. 10.1080/17461391.2016.1188992 27240002

[CR45] Pollak KA, Swenson JD, Vanhaitsma TA, Hughen RW, Jo D, Light KC, Schweinhardt P, Amann M, Light AR (2014) Exogenously applied muscle metabolites synergistically evoke sensations of muscle fatigue and pain in human subjects. Exp Physiol 99:368–380. 10.1113/expphysiol.2013.075812 PMC3946674 24142455

[CR46] Rohel A, Bouffard J, Patricio P, Mavromatis N, Billot M, Roy J-S, Bouyer L, Mercier C, Masse-Alarie H (2021) The effect of experimental pain on the excitability of the corticospinal tract in humans: A systematic review and meta-analysis. Eur J Pain 25:1209–1226. 10.1002/ejp.1746 33565699

[CR47] Sanderson A, Wang SF, Elgueta-Cancino E, Martinez‐Valdes E, Sanchis‐Sanchez E, Liew B, Falla D (2021) The effect of experimental and clinical musculoskeletal pain on spinal and supraspinal projections to motoneurons and motor unit properties in humans: A systematic review. Eur J Pain 25:1668–1701. 10.1002/ejp.1789 33964047

[CR48] Schönbrodt FD, Wagenmakers E-J (2018) Bayes factor design analysis: planning for compelling evidence. Psychon Bull Rev 25:128–142 10.3758/s13423-017-1230-y 28251595

[CR49] Slater M, Banakou D, Beacco A, Gallego J, Macia-Varela F, Oliva R (2022) A separate reality: An update on place illusion and plausibility in virtual reality. Front Virtual Real 3:914392. 10.3389/frvir.2022.914392

[CR50] Steininger MO, White MP, Lengersdorff L, Zhang L, Smalley AJ, Kühn S (2025) Lamm C Nature exposure induces analgesic effects by acting on nociception-related neural processing. Nat Commun. 10.1038/s41467-025-56870-2 PMC11906725 40082419

[CR51] Tamura Y, Okabe S, Ohnishi T, Saito DN, Arai N, Mochio S, Inoue K, Ugawa Y (2004) Effects of 1-Hz repetitive transcranial magnetic stimulation on acute pain induced by capsaicin. Pain 107:107–115. 10.1016/j.pain.2003.10.011 14715396

[CR52] Van Damme S, Crombez G, Van Nieuwenborgh-De Wever K, Goubert L (2008) Is distraction less effective when pain is threatening? An experimental investigation with the cold pressor task. Eur J Pain 12:60–67. 10.1016/j.ejpain.2007.03.001 17433739

[CR53] Richter M, Friedrich A, Gendolla GHE (2008) Task difficulty effects on cardiac activity. Psychophysiol 45:869-875. 10.1111/j.1469-8986.2008.00688.x 18665860

