# Supplementary material for: Nature immersion in virtual reality has limited hypoalgesia effects for muscle pain or motor performance benefits during prescribed and self-regulated tasks
Source: Eur J Appl Physiol. 2026 Aug 19;126(11):6285–301. doi: 10.1007/s00421-026-06399-z (PMC13751703; doi:10.1007/s00421-026-06399-z)
Supplement: Supplementary file 1 [file 421_2026_6399_MOESM1_ESM.pdf]

Supplementary Materials for

Nature immersion in virtual reality has limited hypoalgesia effects for muscle pain or motor performance benefits during prescribed and self-regulated tasks.

Provided within this document are supplementary tables and figures relating to the manuscript noted above. In accordance with open science and research practices, anonymised data from the study is also available at the project's Open Science Framework page (osf.io/eaqbg/) along with the study pre-registrations and preprint copies.

Should you have any questions relating to this manuscript and its content, please do not hesitate to contact the corresponding authors, who's details are provided on the title page of the main manuscript.

Table S1. Participant characteristics, pseudo-randomisation and counterbalancing order for Study 1 protocols

Order Number	Participant	Sex	Age	Height	Mass	Condition Orders		
		M/F	years	cm	kg	Visit		
						1	2	3
1	N1	M	20	175	79	EXP	SHAM	CON
2	N2	F	21	174	69	EXP	CON	SHAM
3	N3	F	23	154	43	SHAM	EXP	CON
4	N4	F	20	169	62	SHAM	CON	EXP
5	N5	F	20	166	54	CON	EXP	SHAM
6	N6	M	21	191	77	CON	SHAM	EXP
7	N7	M	21	178	80	EXP	SHAM	CON
8	N8	M	21	173	73	EXP	CON	SHAM
9	N9	M	21	176	69	SHAM	EXP	CON
10	N10	M	21	181	73	SHAM	CON	EXP
11	N11	F	20	160	55	CON	EXP	SHAM
12	N12	F	20	164	57	CON	SHAM	EXP
Average			20.8	171.8	65.9			
SD			0.8	9.5	11.2			

Legend: M – Male, F – Female; EXP – Experimental; CON - Control

Table S2. Participant characteristics, pseudo-randomisation and counterbalancing order for Study 2 protocols

Order Number	Participant	Sex	Age	Height	Mass	Ethnicity	Handedness	Physical Activity	VISIT 1				VISIT 2			
		M/F	years	cm	kg	UK Categories	R/L	h/week	Fixed Perceived Effort Intensity	Order within Visits			Fixed Perceived Effort Intensity	Order within Visits		
1	N1	F	31	164	59	Asian	R	2	STRONG	EXP	SHAM	CON	LIGHT	EXP	SHAM	CON
2	N2	F	24	170	60	White	R	6	STRONG	EXP	CON	SHAM	LIGHT	EXP	CON	SHAM
3	N3	F	26	169	67	Asian	R	6	STRONG	SHAM	EXP	CON	LIGHT	SHAM	EXP	CON
4	N4	F	19	158	57	White	R	4	STRONG	SHAM	CON	EXP	LIGHT	SHAM	CON	EXP
5	N5	F	22	164	56	Asian	R	5	STRONG	CON	EXP	SHAM	LIGHT	CON	EXP	SHAM
6	N6	F	27	176	82	Asian	R	5	STRONG	CON	SHAM	EXP	LIGHT	CON	SHAM	EXP
7	N7	M	31	174	77	White	L	5	LIGHT	EXP	SHAM	CON	STRONG	EXP	SHAM	CON
8	N8	M	23	180	65	White	R	2	LIGHT	EXP	CON	SHAM	STRONG	EXP	CON	SHAM
9	N9	M	40	162	66	Asian	R	7	LIGHT	SHAM	EXP	CON	STRONG	SHAM	EXP	CON
10	N10	M	31	170	85	White	R	8	LIGHT	SHAM	CON	EXP	STRONG	SHAM	CON	EXP
11	N11	M	25	177	75	White	R	8	LIGHT	CON	EXP	SHAM	STRONG	CON	EXP	SHAM
12	N12	M	29	180	77	White	R	4	LIGHT	CON	SHAM	EXP	STRONG	CON	SHAM	EXP
1	N13	M	29	173	73	White	R	8	STRONG	EXP	SHAM	CON	LIGHT	EXP	SHAM	CON
2	N14	M	33	180	84	White	R	12	STRONG	EXP	CON	SHAM	LIGHT	EXP	CON	SHAM
3	N15	M	31	176	105	White	R	5	STRONG	SHAM	EXP	CON	LIGHT	SHAM	EXP	CON
4	N16	M	22	185	85	Mixed	R	8	STRONG	SHAM	CON	EXP	LIGHT	SHAM	CON	EXP
5	N17	M	28	178	70	White	R	8	STRONG	CON	EXP	SHAM	LIGHT	CON	EXP	SHAM
6	N18	M	26	179	68	White	R	7	STRONG	CON	SHAM	EXP	LIGHT	CON	SHAM	EXP
7	N19	F	28	168	70	Other	R	7	LIGHT	EXP	SHAM	CON	STRONG	EXP	SHAM	CON
8	N20	F	22	160	70	White	R	4	LIGHT	EXP	CON	SHAM	STRONG	EXP	CON	SHAM
9	N21	F	23	173	65	White	R	5	LIGHT	SHAM	EXP	CON	STRONG	SHAM	EXP	CON
10	N22	F	21	168	70	White	R	3	LIGHT	SHAM	CON	EXP	STRONG	SHAM	CON	EXP
11	N23	F	28	173	73	White	R	15	LIGHT	CON	EXP	SHAM	STRONG	CON	EXP	SHAM
12	N24	F	29	173	54	White	R	5	LIGHT	CON	SHAM	EXP	STRONG	CON	SHAM	EXP
Average			27.0	172.1	71.4			6.2								
SD			4.6	6.9	11.2			2.9								

Legend: M – Male F – Female; R – Right; L – Left; EXP – Experimental, CON - Control

Table S3. Outputs for main and interaction effects for (a) muscle pain, (b) force measures, (c) normalised force measures, and (d) contraction duration within Study 2.

a

		Muscle Pain		
		F (df)	p	η_P^2
Main Effects	Intensity	31.274 (1,23)	<.001	.576
	Condition	0.206 (2,46)	.815	.009
	Repetition	19.024 (1.248,28.713)	<.001	.453
Interaction Effect	Intensity x Condition	4.656 (2,46)	.014	.168
	Condition x Repetition	1.874 (2.730,62.786)	.148	.359
	Intensity x Condition x Repetition	1.076 (3.016,69.363)	.365	.045

b

		Peak Force			Mean Force			Force-Time Integral			Peak RFD		
		F (df)	p	η_P^2	F (df)	p	η_P^2 (95%CI)	F (df)	p	η_P^2 (95%CI)	F (df)	p	η_P^2 (95%CI)
Main Effect	Intensity	100.984 (1,23)	<.001	.814	91.129 (1,23)	<.001	.798	90.541 (1,23)	<.001	.797	79.286 (1,23)	<.001	.775
	Condition	0.496 (2,46)	.612	.021	0.710 (2,46)	.497	.030	1.150 (2,46)	.325	.048	2.891 (2.46)	.066	.112
	Repetition	45.691 (1.799,41.382)	<.001	.665	43.588 (1.923,44.223)	<.001	.655	43.615 (2.147,49.375)	<.001	.655	10.085 (1.615,37.154)	<.001	.305
Interaction Effects	Intensity x Condition	0.339 (2,46)	.715	.015	0.155 (2,46)	.856	.007	0.066 (2,46)	.937	.003	0.778 (2,46)	.465	.033
	Condition x Repetition	0.711 (6,138)	.641	.030	1.011 (6.138)	.421	.042	1.117 (6,138)	.356	.046	2.358 (6,138)	.034	.093
	Intensity x Condition x Repetition	0.689 (6,138)	.659	.029	0.954 (6,138)	.459	.040	1.342 (6.138)	.243	.055	1.446 (6,138)	.201	.059

c

		Normalised Peak Force			Normalised Peak RFD		
		F (df)	p	η_P^2 (95%CI)	F (df)	p	η_P^2 (95%CI)
Main Effects	Intensity	142.887 (1,23)	<.001	.861	172.114 (1,23)	<.001	.882
	Condition	0.223 (2,46)	.801	.010	1.369 (1.484,34.124)	.263	.056
	Repetition	40.101 (1.871,43.040)	<.001	.636	7.726 (1.637,37.651)	.003	.251
Interaction Effect	Intensity x Condition	2.804 (1.534,35.276)	.087	.109	0.307 (2,46)	.737	.013
	Condition x Repetition	0.605	.726	.026	2.824	.024	.109

		(6,138)			(6,138)		
	Intensity x Condition x Repetition	0.421 (6,138)	.864	.018	1.021 (6,138)	.414	.043

d		Contraction Duration		
		F (df)	p	η_p^2 (95%CI)
Main Effects	Intensity	0.007 (1,23)	.933	<.001
	Condition	1.789 (2,46)	.186	.072
	Repetition	1.074 (2.071,47.622)	.352	.045
Interaction Effect	Intensity x Condition	0.300 (2,46)	.742	.013
	Condition x Repetition	1.368 (2.968,68.268)	.260	.056
	Intensity x Condition x Repetition	3.126 (6,138)	.007	.120

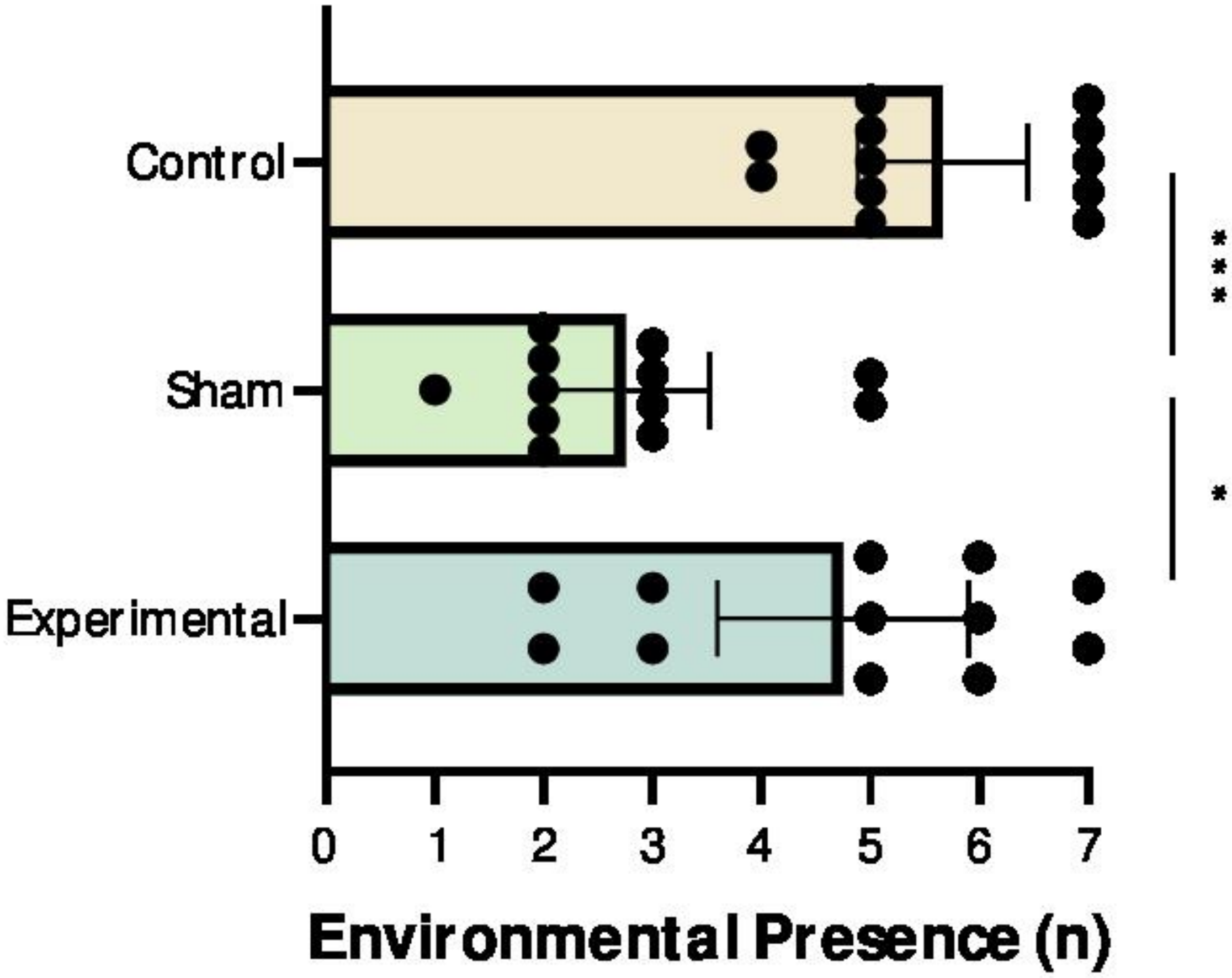

Figure S1. Study 1 environmental presence ratings. Individual Plots represent individual data. Error bars represent standard deviations. ∗ = p < .05, ∗∗∗ = p < .001.

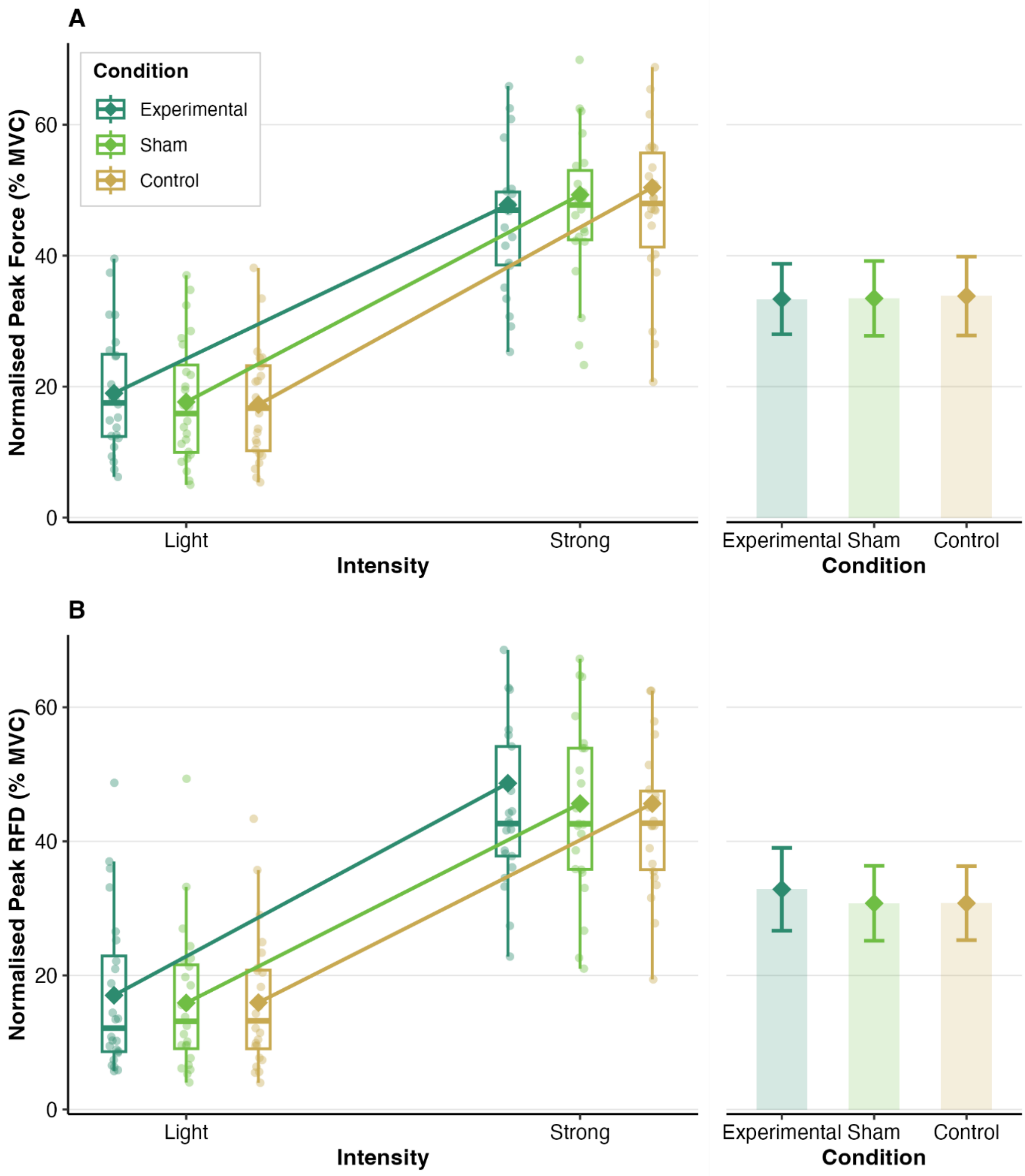

Figure S2. Normalised peak force (A) and peak rate of force development (RFD) (B) from Study 2.

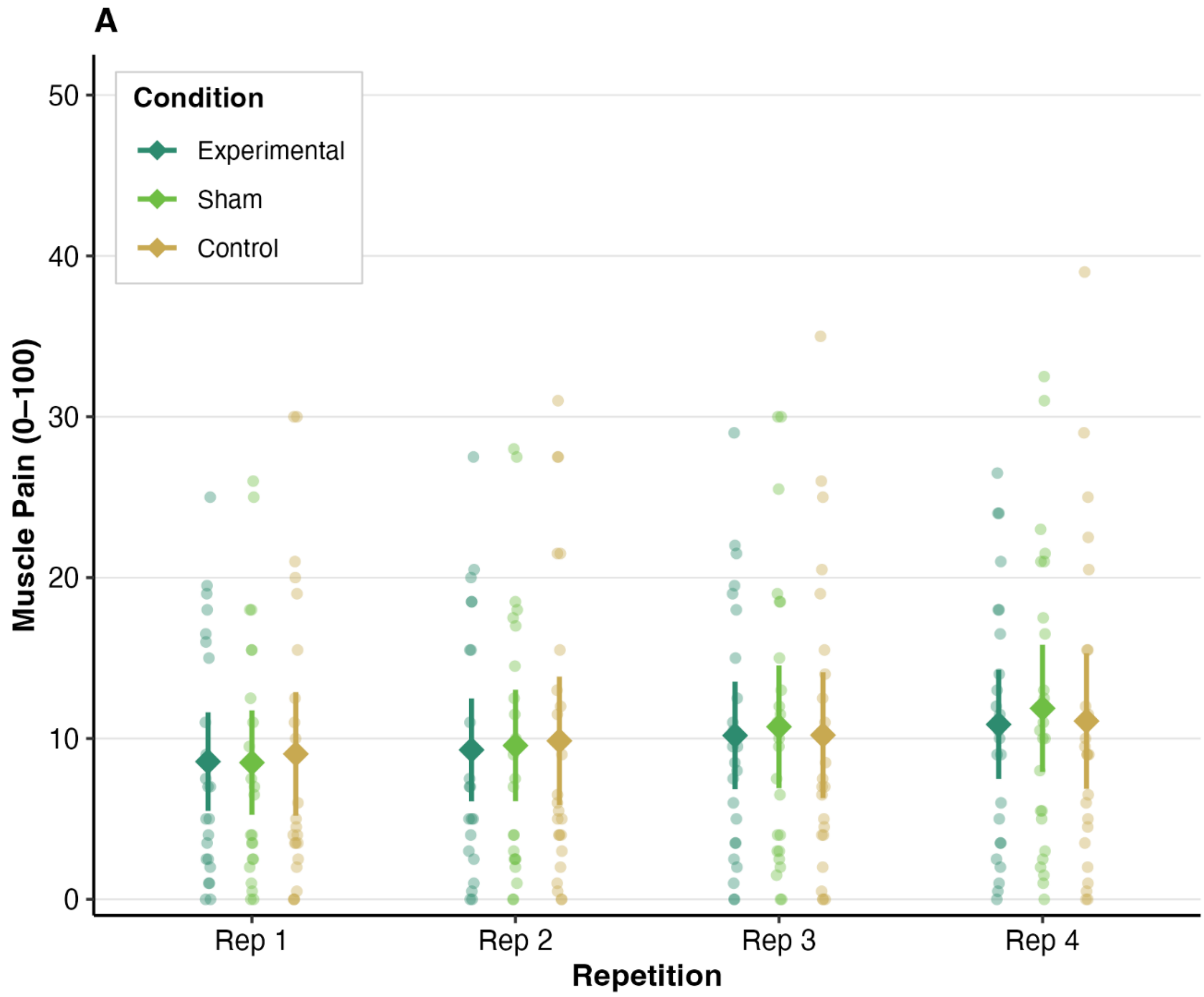

Figure S3. Naturally occurring muscle pain changes over each repetition in Study 2.

Muscle pain showed notable increases across repetitions ($F_{1.248,28.713} = 19.024, p < .001, \eta_p^2 = 0.453[0.165, 0.623]$) with pairwise comparisons showing a progressive increase in muscle pain ratings through each repetition ($t_{23} = -2.985 - 4.704, p's = .001 - .040$).

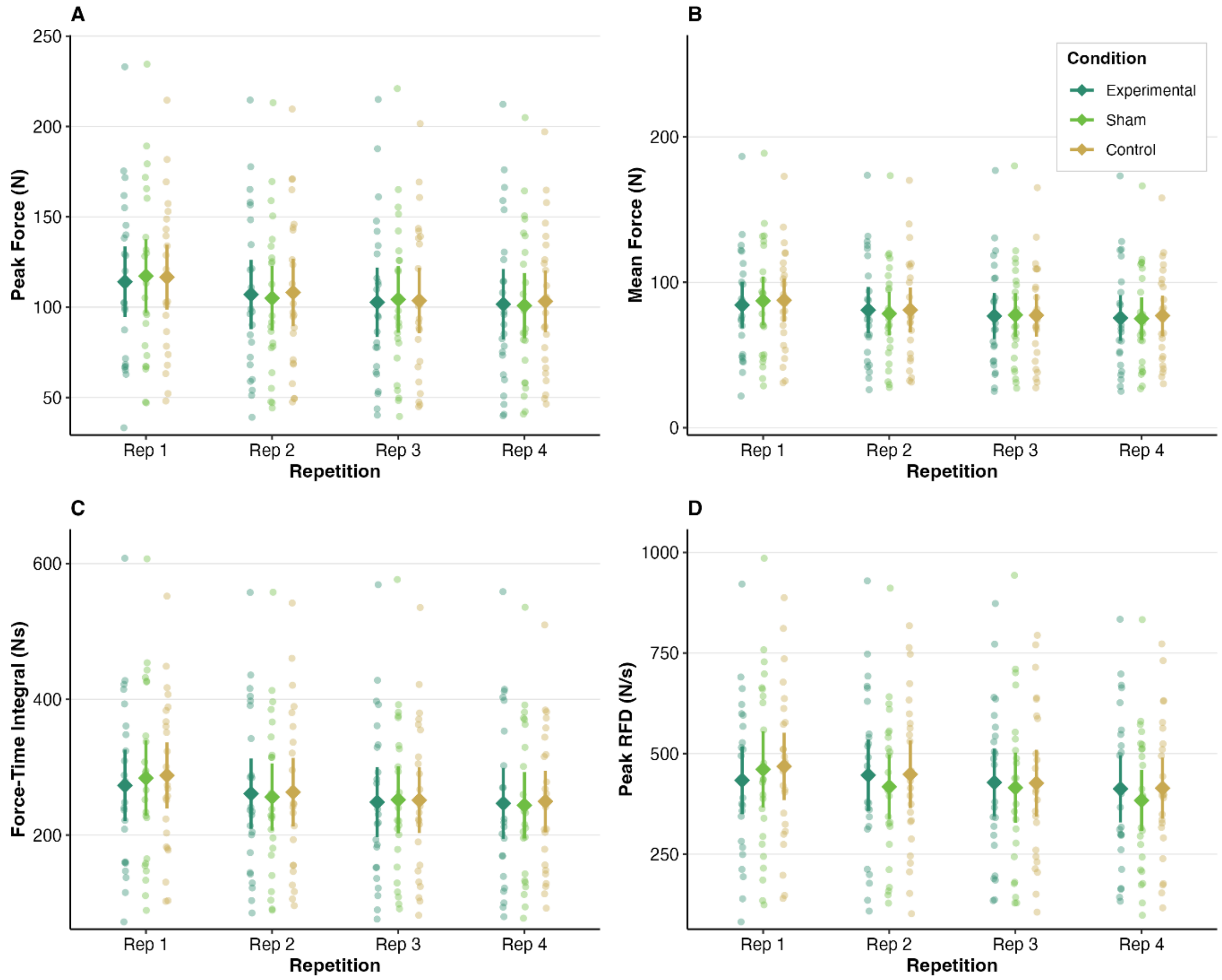

Figure S4. Peak force (A), mean force (B), force-time integral (C), and peak rate of force development (RFD) (D) changes over each condition in Study 2.

Peak force ($F_{1.799,41.382} = 45.691, p < .001, \eta_p^2 = 0.665[0.467,0.758]$), mean force ($F_{1.923,44.223} = 43.588, p < .001, \eta_p^2 = 0.655[0.459,0.748]$), force-time integral ($F_{2.147,49.375} = 43.615, p < .001, \eta_p^2 = 0.655[0.471,0.743]$), and peak RFD ($F_{1.615,37.154} = 10.085, p < .001, \eta_p^2 = 0.305[0.070,0.485]$) all showed notable declines across repetitions. Normalised peak force ($F_{1.871,43.040} = 40.101, p < .001, \eta_p^2 = 0.636[0.430,0.735]$) and normalised peak RFD ($F_{1.637,37.651} = 7.726, p = .003, \eta_p^2 = 0.251[0.038,0.436]$) also showed notable declines across repetitions. Pairwise comparisons showed that peak force, mean force, force-time integral, and normalised peak force was higher at repetition 1 versus repetitions 2 ($t_{23} = 4.997 - 5.808, p's < .001$), 3 ($t_{23} = 7.383 - 8.160, p's =< .001$), and 4 ($t_{23} = 7.562 - 8.504, p's < .001$), with an eventual plateau in force measures between repetitions 3 and 4 ($p's > .274$).

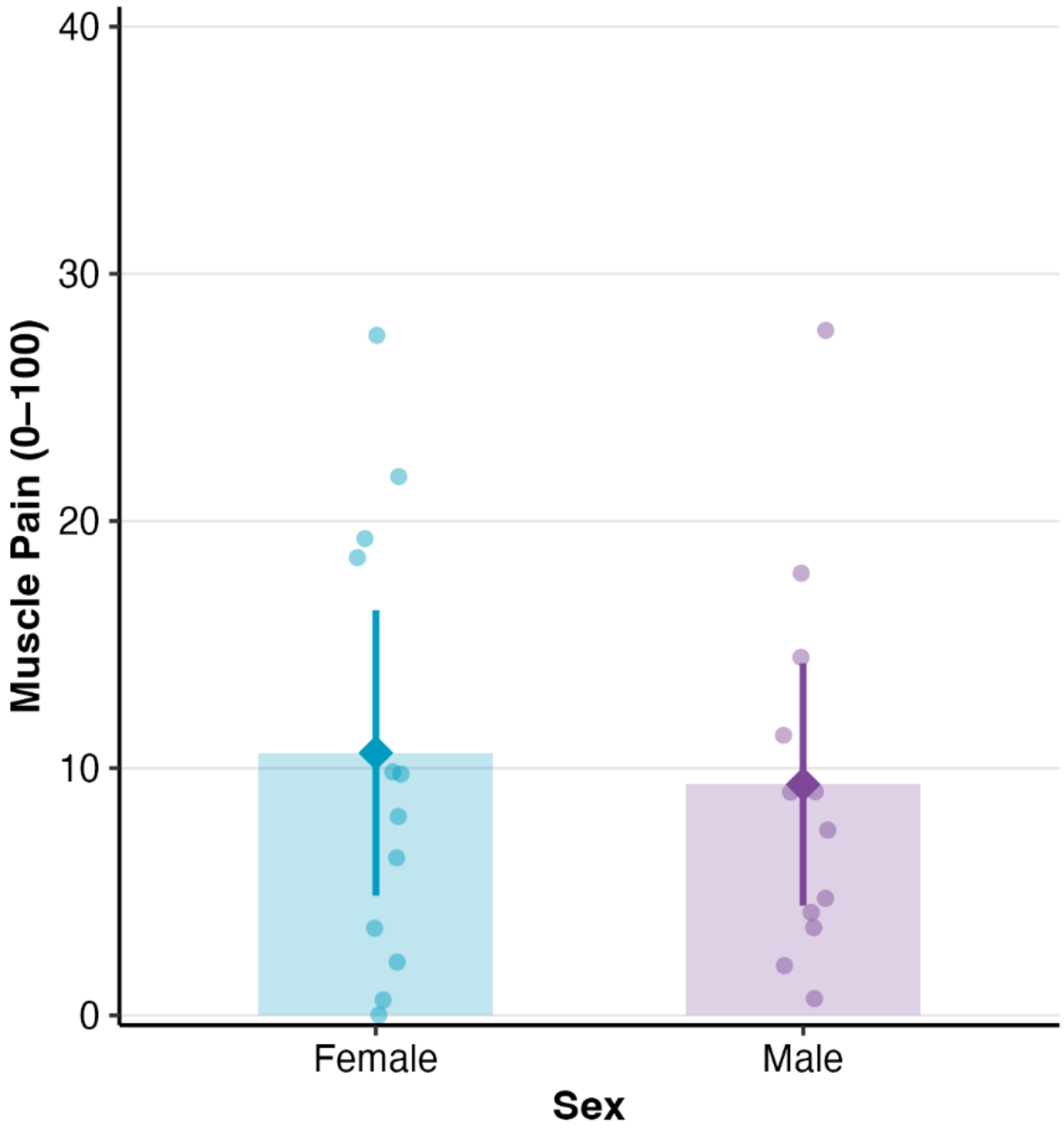

Figure S5. Mean naturally occurring muscle pain ratings by sex in Study 2.

Exploratory analysis showed no difference in muscle pain ratings between sexes ($U = 2370, p = .413, r = 0.079$).

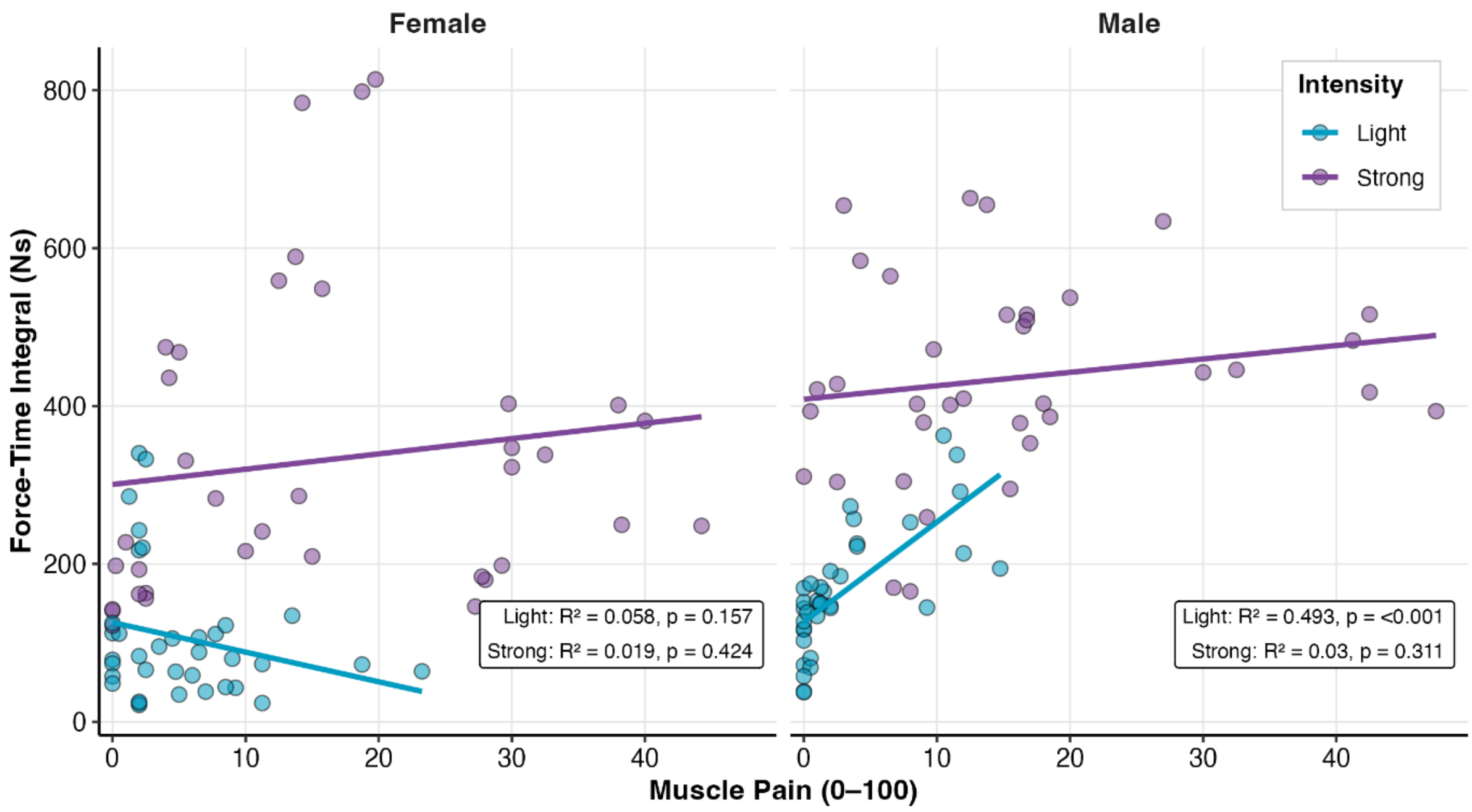

Figure S6. Study 2 linear regression assessing changes in force production (force-time integral) with naturally occurring muscle pain changes with sexes separated.

The first regression analysis revealed that muscle pain ratings had a positive effect (i.e., increase) on measures of force production (force-time integral) ($t = 5.631, p < .001$). When adding intensity as a factor, there was a main effect of intensity ($t = 6.954, p < .001$). However, the effect of muscle pain was no longer significant ($p = .724$). For each one-unit increase in muscle pain ratings, peak force still increased force-time integral by 1.13 N·s. The interaction between intensity and muscle pain was not significant on force-time integral ($p = .854$), meaning that force increases were similar between intensities. Males exerted a higher peak force ($U = 1791, p = .001. r = 0.309$), mean force ($U = 1759, p = .001. r = 0.321$), and force-time integral ($U = 1745, p = .001. r = 0.327$) than females. The third model of the first exploratory linear regression analysis showed a muscle pain × intensity × sex interaction ($t = -2.459, p = .015$). The effect of muscle pain was not significant ($p = .319$). Main difference in force production between sexes were in the light intensity, where males seemed to increase force production as muscle pain increased whereas females decreased force production.

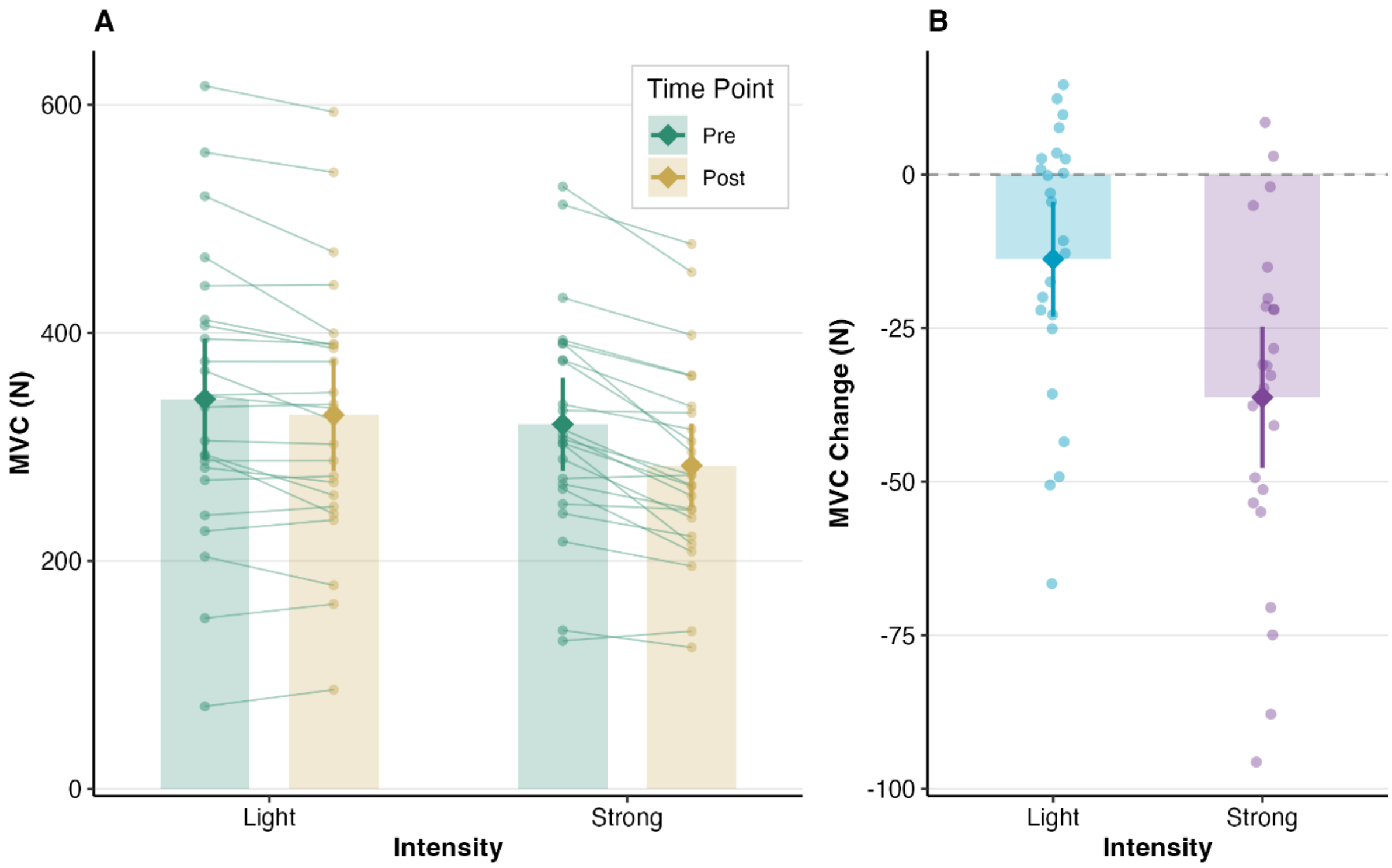

Figure S7. Study 2 maximal voluntary contraction (MVC) peak force changes from pre to post condition (A) and averaged for all conditions across light and strong intensities (B).

Pre-post condition peak force during the MVC decreased more in the strong versus the light condition ($t_{23} = 4.335, p < .001, d = 0.885[0.404 - 1.352]$), indicating a greater degree of fatigue occurred in the strong versus light intensity as expected. There were no differences in MVC peak force changes between conditions ($F_{2,46} = 0.820, p = .447, \eta_p^2 = 0.034[0.000, 0.154]$).

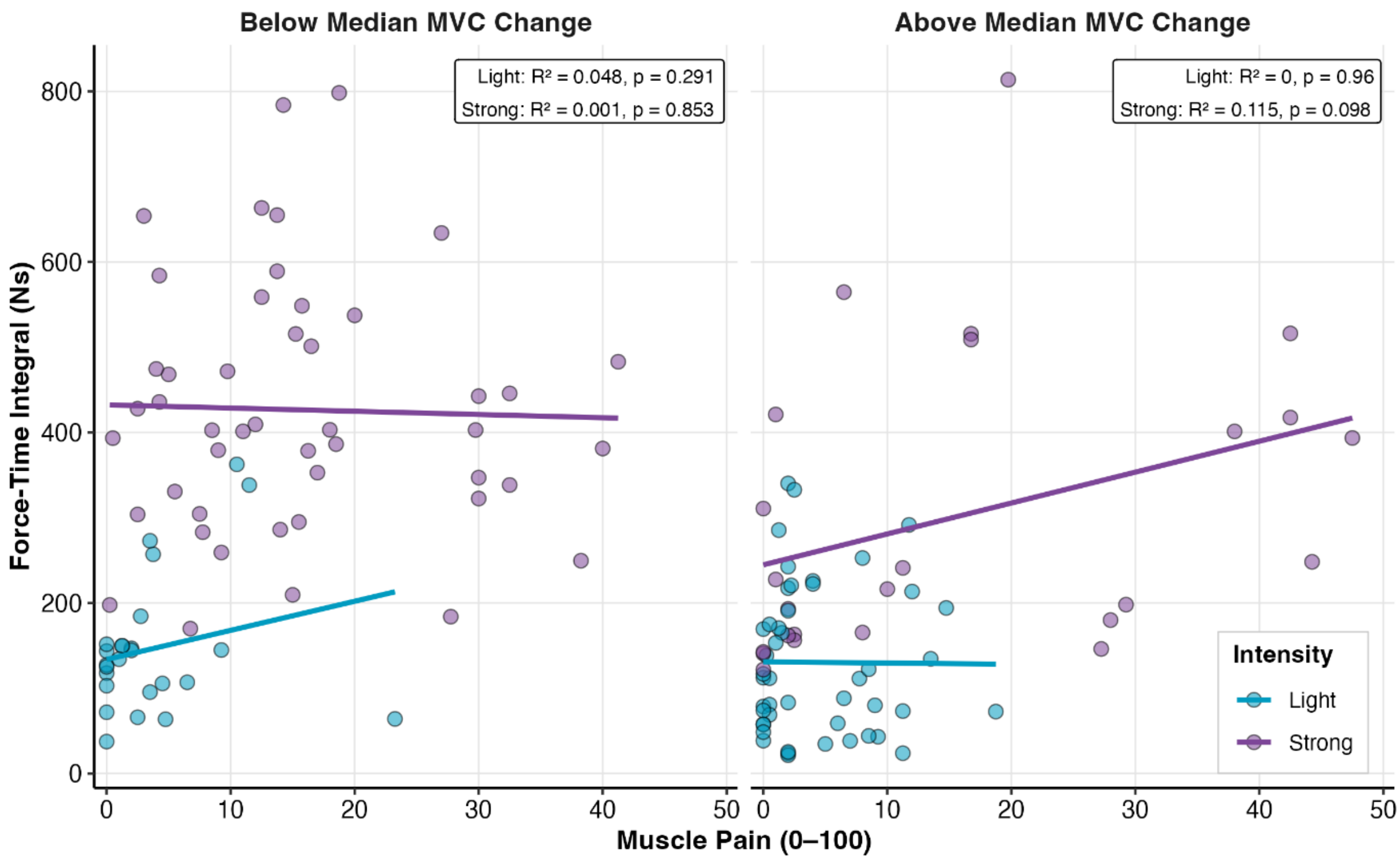

Figure S8. Study 2 linear regression assessing changes in force production (force-time integral) with naturally occurring muscle pain changes with objective fatigue (change in peak force from pre-post conditions maximal voluntary contractions) separated.

The third linear regression analysis revealed similar results to the first analysis in models one and two. In model three with the inclusion of changes in MVC peak force as a measure of objective fatigue, there was no interaction between muscle pain × intensity × MVC changes (p = .484) and no effect of muscle pain (p = .881) on force production. As objective fatigue increased (i.e., greater changes in peak force from pre to post condition), participants seemed to increase their force production more in the strong intensity as muscle pain increased, whereas in the light intensity less objective fatigue was linked with increases in force production as muscle pain increase.

Supplementary Material – Item S9.

The second linear regression analysis revealed the same results as the first analysis in models one and two. In model three with the inclusion of environmental presence ratings, there was no interaction between muscle pain × intensity × presence ratings ($p = .143$) and no effect of muscle pain ($p = .089$) on force production.
